# Investigation into the Pancreatic Pathogenesis of SFTSV across Multiple Levels

**DOI:** 10.1002/advs.202515862

**Published:** 2025-10-15

**Authors:** Xiaohan Liu, Zhihao Xu, Yilun Tong, Changtai Wang, Yueqi Yao, Yujie Diao, Jingyuan Ma, Shijun Zhou, Yinan Du, Zhenhua Zhang, Gang Xu

**Affiliations:** ^1^ School of Basic Medical Sciences Anhui Medical University Hefei 230032 China; ^2^ The First Clinical Medical School Anhui Medical University Hefei 230032 China; ^3^ School of Basic Medical Sciences Tongji Medical College, Huazhong University of Science and Technology Wuhan 430030 China; ^4^ Department of Infectious Diseases The Second Affiliated Hospital of Anhui Medical University Hefei 230061 China; ^5^ Department of Blood Transfusion The First Affiliated Hospital of Anhui Medical University Hefei 230022 China

**Keywords:** pancreatic tropism, severe fever with thrombocytopenia syndrome virus, tick‐borne infectious diseases, viral hemorrhagic fever, viral pancreatitis

## Abstract

Severe Fever with Thrombocytopenia Syndrome virus (SFTSV) infection induces hepatitis, myocarditis, and even multi‐organ dysfunction with a high mortality rate, while the impact on the pancreas remains unknown. In a retrospective analysis of clinical parameters in a cohort of 290 patients with severe fever with thrombocytopenia syndrome (SFTS), it is observed that pancreatic injury biomarkers in 19.4% (elevated serum amylase ≥3 × upper limit of normal (ULN)) and 25.8% (elevated serum lipase ≥3 × ULN). Notably, 17.6% of patients met the diagnostic criteria for clinically confirmed pancreatitis. Mechanistic studies using human pancreatic organoids and murine models demonstrated that SFTSV directly infects pancreatic tissue, facilitated by viral receptors C‐C motif chemokine receptor 2 (CCR2) and lipoprotein receptor‐related protein 1 (LRP1), provoking cell death in pancreatic tissue. Transcriptomic profiling revealed that SFTSV infection triggers a robust innate immune response characterized by interferon pathway activation and pro‐inflammatory cytokine upregulation. Pathological analysis of infected murine pancreatic tissues showed acinar cell vacuolization, viral inclusions, and immune cell infiltration. Comparative studies with caerulein‐induced pancreatitis models identified C3‐mediated complement hyperactivation as a key pathological driver. The studies identified that SFTSV exhibits a specific pancreatic tropism, with direct infection leading to cell death and initiating a strong inflammatory immune response, resulting in viral pancreatitis.

## Graphical abstract

A retrospective analysis revealed that 17.6% of severe fever with thrombocytopenia syndrome (SFTS) patients met the diagnostic criteria for clinically confirmed pancreatitis. Using human pancreatic organoids and murine models demonstrated that SFTSV exhibits a specific pancreatic tropism, leading to cell death and initiating a strong inflammatory immune response, resulting in viral pancreatitis. Comparative studies identified C3‐mediated complement hyperactivation as a key pathological driver.

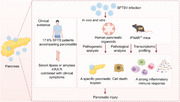

## 1 Introduction

Severe fever with thrombocytopenia syndrome (SFTS), an emerging tick‐borne zoonosis first identified in 2009, is caused by severe fever with thrombocytopenia syndrome virus (SFTSV), a member of the *Bandavirus* genus within the *Phenuiviridae* family of the order *Bunyavirales*.^[^ ^1^ ^]^ Over recent decades, SFTS has not only spread to most provinces in China, but also poses a threat to East and Southeast Asia.^[^ ^2^, ^3^, ^4^, ^5^, ^6^ ^]^ Critically, the incidence of SFTS demonstrates a concerning upward trajectory year by year with case fatality rates of 7.80%,^[^ ^7^ ^]^ prompting its designation by the World Health Organization (WHO) in 2017 as a priority disease.^[^ ^8^ ^]^ Currently, there is a severe shortage of effective antivirals and approved vaccines due to our poor understanding of the pathogenesis, which greatly hampers patient management and outbreak control.

SFTSV infection exhibits heterogeneous clinical manifestations, ranging from self‐limiting febrile illness to life‐threatening systemic complications.^[^ ^9^ ^]^ The major clinical manifestations of SFTS include fever, thrombocytopenia, leukocytopenia, regional lymphadenopathy, myalgia and gastrointestinal symptoms, with most cases having a favorable prognosis.^[^ ^9^, ^10^ ^]^ However, elderly individuals, individuals with underlying health conditions, or those who delay seeking medical attention tend to have more severe symptoms,^[^ ^9^, ^11^ ^]^ and some of the critically ill even die due to multiple organ failure.^[^ ^9^ ^]^ Common laboratory findings include leukocytopenia, thrombocytopenia, elevated levels of aspartate aminotransferase (AST), alanine aminotransferase (ALT), lactic acid dehydrogenase (LDH), and creatine kinase (CK), reflecting immunological dysfunction, liver inflammation, and myocarditis in SFTS patients.^[^ ^9^ ^]^ Although the precise mechanisms underlying multi‐organ injury in SFTS remain incompletely understood, emerging evidence indicates that B cells at diverse differentiation stages toward plasmablasts disseminate from lymphoid reservoirs into non‐lymphoid organs, where they may serve as functional viral reservoirs for SFTSV replication.^[^ ^12^ ^]^

Here, we report a different mechanism of SFTSV‐induced pancreatic injury mediated through direct viral infection. A retrospective analysis of clinical parameters in a cohort of 290 patients with SFTS revealed abnormally elevated amylase and lipase levels, which were positively correlated with viral load. In vitro, pancreatic organoid model studies have shown that SFTSV directly infects pancreatic cells supported by viral receptors and triggered pancreatic cell apoptosis. Murine models further confirmed SFTSV tropism for pancreatic tissue, with obvious pathological features such as acinar cell vacuolation, viral inclusion bodies, and immune cell infiltration. Compared to caerulein‐induced pancreatitis models, SFTSV infection provoked a stronger innate immune response characterized by high expression of inflammatory cytokines. Crucially, both models simultaneously exposed C3‐driven complement hyperactivation, uncovering the pivotal role of the complement‐mediated inflammatory axis in pancreatitis pathology. This study clearly demonstrates the mechanism of pancreatic injury caused by SFTSV infection: direct viral infection of the pancreas induces immune responses such as cell death, complement activation, inflammation, etc., thereby significantly advancing our understanding of the pathogenesis of SFTS.

## 2 Results

### 2.1 SFTSV Infection Induces Viral Pancreatitis

**Figure 1:**
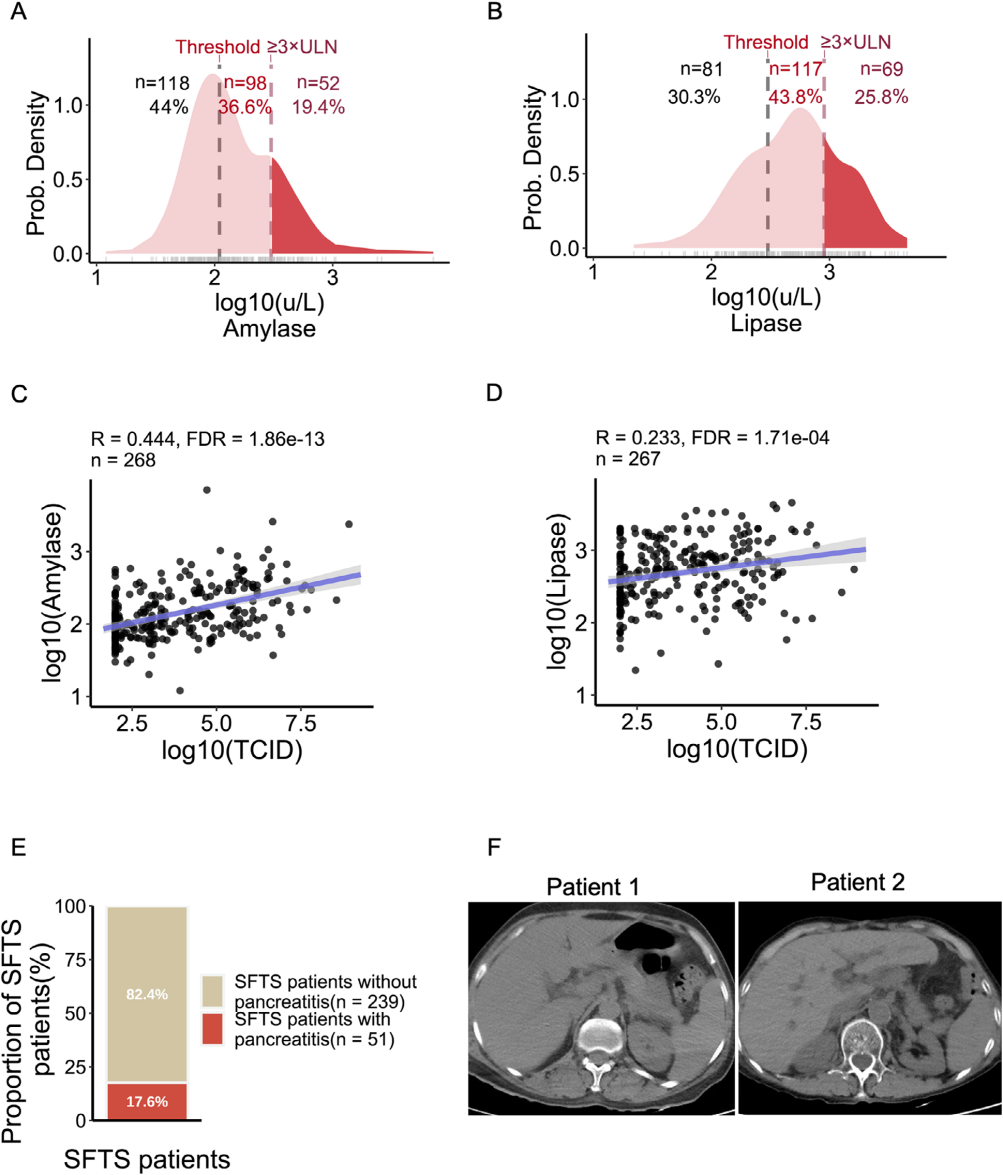
Clinical cohorts demonstrated a close association between SFTSV infection and pancreatitis. A) Probability density distribution and proportion of amylase concentration in peripheral blood of SFTS patients (*n* = 268). The amylase concentration is log10‐transformed, and the upper limit of normal (ULN) for amylase is 110 UL^−1^. B) Probability density distribution and proportion of lipase concentration in peripheral blood of SFTS patients (*n* = 267). The lipase concentration is log10‐transformed, and the ULN for lipase is 300 UL^−1^. C) Scatter plot with regression of viral load of SFTS patients and amylase concentration. The solid line represents the best‐fit linear regression line, and the shaded area is the 95% confidence interval. Pearson correlation coefficient indicates a significant positive correlation between the two variables (*R* = 0.444, FDR<0.05, *n* = 268). D) Scatter plot with regression of viral load of SFTS patients and lipase concentration. The solid line represents the best‐fit linear regression line, and the shaded area is the 95% confidence interval. Pearson correlation coefficient indicates a significant positive correlation between the two variables (*R* = 0.233, FDR<0.05, *n* = 267). E) The number and proportion of SFTS patients who presented with symptoms of pancreatitis. F) CT images of SFTS patients did not exhibit typical features of pancreatitis.

A retrospective analysis of clinical data from 290 SFTS patients from the First and Second Affiliated Hospitals of Anhui Medical University confirmed previously reported abnormalities including lymphocytopenia, thrombocytopenia, elevated levels of AST, ALT, LDH, and cytokines (IL‐6, IL‐1β, TNF‐α) (Table S1, Supporting Information). Notably, amylase and lipase levels were significantly elevated outside the normal ranges (Table S1, Supporting Information), a phenomenon rarely reported previously. Specifically, 56% of patients had amylase levels exceeding the upper limit of normal (ULN), with 19.4% exceeding 3 × ULN thresholds (**Figure** **1**A), while 69.6% of patients had lipase levels exceeding the ULN with 25.8% surpassing 3 × ULN the alert value (Figure 1B). Strikingly, amylase and lipase levels demonstrated positive correlations with viral load but not with age or sex (Figure 1C,D; Figure S1A,B, Supporting Information). Amylase and lipase are the main early warning biomarkers of pancreatic injury.^[^ ^13^ ^]^ Combined with clinical symptoms such as persistent abdominal pain, 17.6% of the patients were diagnosed with viral pancreatitis (Figure 1E). Compared to SFTS patients without pancreatitis, those with pancreatitis exhibited significantly elevated amylase and lipase levels, along with higher viral loads (Figure S1C,D, Supporting Information). However, computed tomography (CT) images of these patients do not show typical abnormalities such as pancreatic enlargement, inflammatory exudation, peripancreatic fluid accumulation, and potential pancreatic parenchymal necrosis (Figure 1F; Figure S1E, Supporting Information), which is significantly different from pancreatitis caused by gallstones or chronic alcohol intake.^[^ ^14^, ^15^ ^]^ Collectively, these results indicate that SFTSV infection can cause pancreatic damage in patients and is positively correlated with viral load.

### 2.2 SFTSV Directly Infects Pancreatic Organoids and Activates Robust Innate Immunity

**Figure 2:**
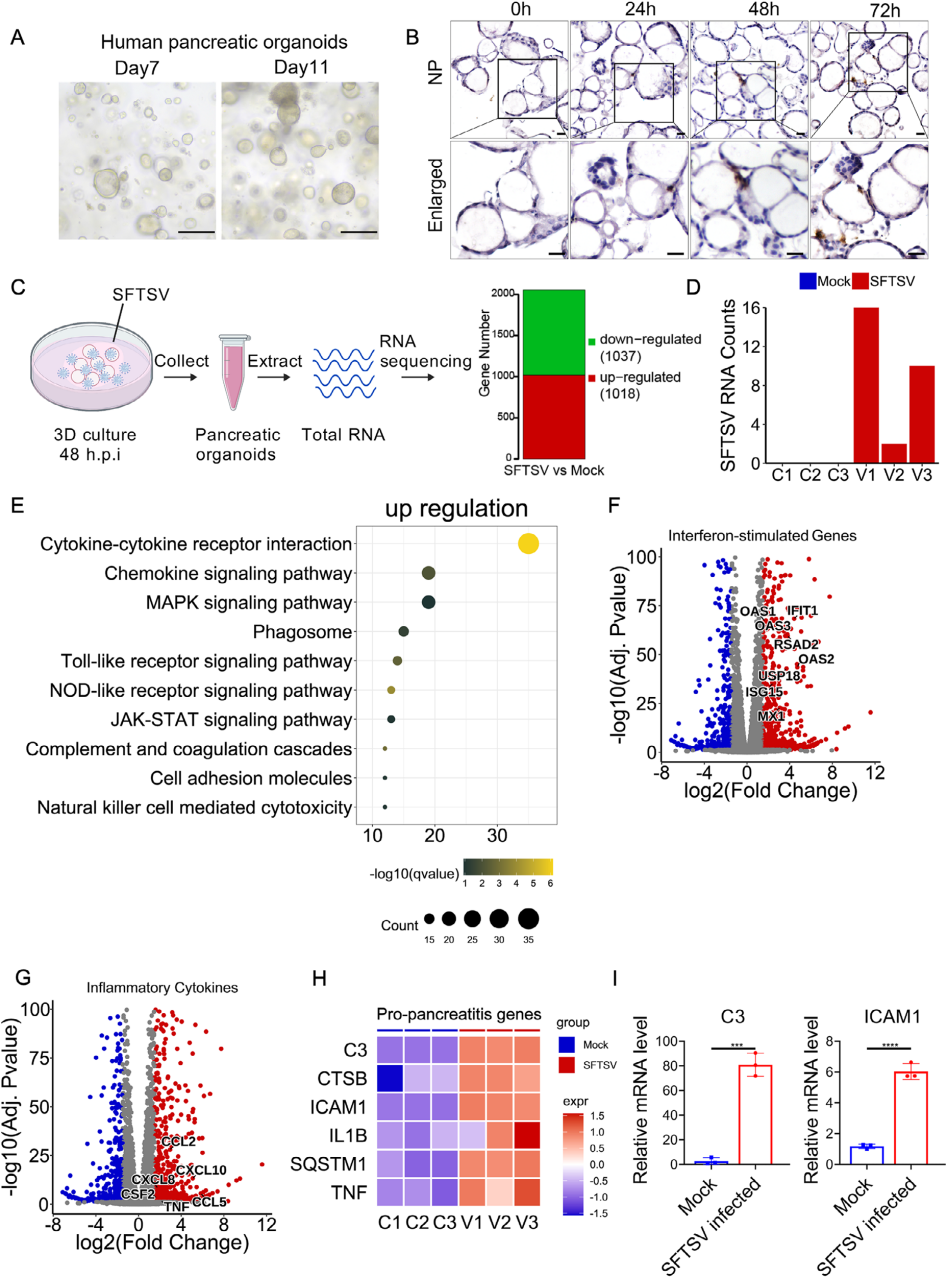
Direct infection of pancreatic organoids by SFTSV triggers robust innate immune responses. A) Bright‐field morphology of human pancreatic organoids at different time points of culture. Scale bar, 200 µm. B) Immunohistochemical detection of NP expression in human pancreatic organoids at different time points following mock and SFTSV infection, scale bars, 20 µm. C) RNA was extracted from human pancreatic organoids infected with SFTSV for 48 h and from the mock group for transcriptome sequencing, with three biological replicates per group (created with BioGDP.com). D) The counts of SFTSV genomic fragments detected in each sample. E) The bubble plot shows the top 10 terms in the KEGG analysis of upregulated genes in pancreatic organoids of SFTSV infection. The volcano plot shows the representative interferon‐stimulated genes. F) and the representative inflammatory cytokine‐related genes G) among the upregulated genes. H) Heatmap displaying the expression of pro‐pancreatitis genes in RNA‐seq of human pancreatic organoids, where gene expression values are replaced by normalized relative expression values. I) qRT‐PCR validation of *C3* and *ICAM1* relative mRNA expression levels from the pro‐pancreatitis genes heatmap in human pancreatic organoid RNA‐seq. Experiment was performed in three biological replicates with similar results. Data shown are means ± SEM. Statistical significance was analyzed by Student's t‐test. *p < 0.05; **p < 0.01; ***p < 0.001; **** p < 0.0001.

To investigate SFTSV tropism for pancreatic tissue, we constructed 3D human pancreatic organoids composed of ductal cells with reference to Boj et al. ’s study ^[^ ^16^ ^]^ (**Figure** **2**A; Figure S2A, Supporting Information). In SFTSV infected organoids, hematoxylin and eosin (H&E) staining showed no significant structural alterations, such as organoid rupture or cell shedding (Figure S2B, Supporting Information). To depict the SFTSV infection scenario, viral replication was confirmed by the robust expression of viral proteins and detection of viral RNA, with the earliest detection occurring 24 h post‐infection (Figure 2B; Figure S2C,D, Supporting Information). Expression of SFTSV receptors C‐C motif chemokine receptor 2 (CCR2) ^[^ ^17^ ^]^ and lipoprotein receptor‐related protein 1 (LRP1) ^[^ ^18^ ^]^ in pancreatic organoids supported viral infection (Figure S2E, Supporting Information). Terminal transferase‐mediated dUTP nick‐end labeling (TUNEL) analysis demonstrated significant cell death in pancreatic organoids, correlating with SFTSV replication (Figure S2F, Supporting Information). These findings collectively suggest pancreatic tropism of SFTSV.

To characterize host response, we performed comprehensive transcriptomic profiling of SFTSV‐infected human pancreatic organoids (Figure 2C). Principal component analysis (PCA) revealed significant transcriptomic divergence between infected and mock organoids (Figure S3A, Supporting Information). Differentially expressed genes (DEGs) were identified using thresholds of |log_2_ fold‐change| ≥ 1 and adjusted *p*‐value < 0.05. After quality control, a total of 1018 genes were upregulated and 1037 genes were downregulated (Figure 2C). Viral transcripts were exclusively detected in infected organoids (Figure 2D). Kyoto Encyclopedia of Genes and Genomes (KEGG) enrichment analysis of upregulated DEGs demonstrated significant activation of innate immune pathways including cytokine‐chemokine signaling, Toll‐like receptor (TLR) signaling, NOD‐like receptor (NLR) signaling and MAPK signaling pathways (Figure 2E). While KEGG enrichment analysis of downregulated DEGs indicated deficient cell cycle regulation and amino acid metabolism (Figure S3B, Supporting Information). We further examined the DEGs associated with those KEGG terms. Indeed, many canonical IFN‐stimulating genes (ISGs), including *ISG15, IFIT1, OAS1, RSAD2*, and *MX1*, etc., were expressed at higher levels in SFTSV infected group than mock group (Figure 2F). Similarly, proinflammatory cytokines *CCL2, CXCL10*, and *CCL5*, etc., were markedly elevated in the infection group (Figure 2G). The higher expression of these ISGs and cytokines in the infection group were confirmed by qRT‐PCR (Figure S3C,D, Supporting Information). Consistent with the previous TUNEL staining results (Figure S2F, Supporting Information), cell death‐related genes were significantly upregulated in infected organoids (Figure S3E, Supporting Information). These findings demonstrate that SFTSV infection triggers a robust innate immune response in pancreatic organoids.

Previous studies have established that intrapancreatic trypsinogen activation mediated by Cathepsin‐B (CTSB), leads to autodigestion and promotes pancreatic damage.^[^ ^19^ ^]^ P62‐driven autophagy‐dependent ferroptosis,^[^ ^20^ ^]^ Complement activation‐induced acinar cell damage ^[^ ^21^, ^22^, ^23^, ^24^ ^]^ and the recruitment of immune cells through cytokines *IL‐1B, TNF*, and adhesion molecule *ICAM1* ^[^ ^25^, ^26^, ^27^ ^]^ have been reported as crucial mechanisms in pancreatitis. The serum level of its soluble form (sICAM‐1) serves as a prognostic biomarker for severe pancreatitis.^[^ ^28^ ^]^ Notably, transcriptomic profiling of SFTSV‐infected pancreatic organoids revealed significant upregulation of these pro‐pancreatitis genes (*C3, CSTB, ICAM1, IL1B, SQSTM1*, and *TNF*) (Figure 2H,I), suggesting that SFTSV likely drives pancreatic injury through these conserved pathogenic pathways.

### 2.3 SFTSV Direct Infection of Murine Pancreas Triggers Inflammatory Cytokine Storm

**Figure 3:**
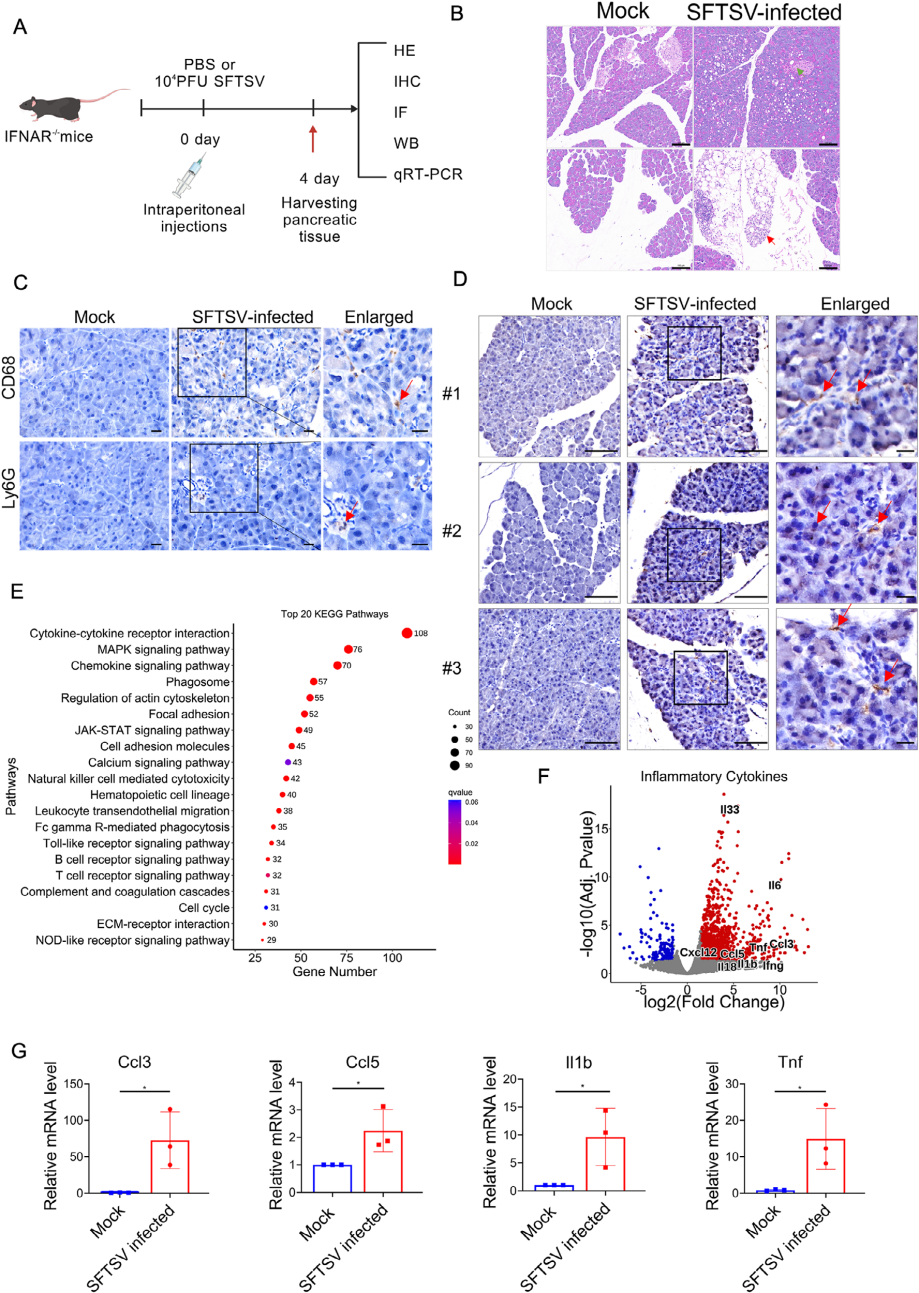
Investigation into the pancreatic tropism and pancreatic injury mechanisms of SFTSV in mouse model. A) Schematic of SFTSV infection in IFNAR^−/−^ mice including mock group (*n* = 3) and SFTSV‐infected group (*n* = 3) received intraperitoneal PBS and 10⁴ PFU virus, respectively. Mice were euthanized on day 4 post‐infection to harvest pancreatic tissues for subsequent analysis. B) The histopathological changes in pancreatic tissues were evaluated by H&E staining. Acinar cell degranulation (green arrow) and inflammatory cell infiltration (red arrow) were showed in the pancreatic sections. Scale bars, 100 µm. C) Immunohistochemical detection of macrophages (CD68, red arrow) and neutrophils (Ly‐6G, red arrow) in pancreatic tissues of SFTSV‐infected and mock‐treated mice, Scale bars, 20 µm. D) Immunohistochemical detection of SFTSV protein NP (red arrows) in pancreatic tissues, Scale bar, 100 and 20 µm in the enlarged image. E) The bubble plot shows the top 20 terms in the KEGG analysis of upregulated genes in pancreatic tissues of SFTSV‐infected mice. F) The volcano plot shows the representative inflammatory cytokine‐related genes among the upregulated genes in pancreatic tissues of SFTSV‐infected mice. G) qRT‐PCR validation of *Ccl3, Ccl5, Il‐1b*, and *Tnf* relative mRNA expression level. Experiment was performed in triplicates. Data shown are means ± SEM. Statistical significance was analyzed by Student's t‐test. *p < 0.05; **p < 0.01; ***p < 0.001; **** p < 0.0001.

To further dissect the mechanism underlying SFTSV‐induced pancreatitis, we investigated the pancreatic tropism, pathological damage, and immune response of SFTSV in interferon receptor knockout (IFNAR^−/−^) mice (**Figure** **3**A). SFTSV‐infected IFNAR^−/−^ mice exhibited significant pancreatic atrophy compared to controls (Figure S4A,B, Supporting Information). The pathological damage phenomena such as degranulation of pancreatic acinar cells, vacuolization of cytoplasm, and narrowing of lobular septa are also quite evident (Figure 3B), along with lymphocytic infiltration containing occasional neutrophils and macrophage (Figure 3C). Moreover, H&E analysis revealed putative viral inclusion body‐like structures within infected pancreatic tissues, indicating active viral replication (Figure S4C, Supporting Information). In fact, Consistent with organoid model studies, viral proteins and RNA were detected in pancreatic tissue (Figure 3D; Figure S4D–F, Supporting Information), confirming the pancreatic tropism of SFTSV. Viral receptors LRP1 and CCR2 were also expressed in murine pancreas (Figure S4G, Supporting Information). Crucially, productive replication was confirmed by isolating infectious virus from pancreatic homogenates of infected mice inoculated onto Vero E6 cells (Figure S5A, Supporting Information), with significantly higher viral titers than the mock group (Figure S5B,C, Supporting Information). Overall, these results demonstrate that SFTSV infects the pancreatic tissue of IFNAR^−/−^mice and replicates robustly within it.

Additionally, to systematically characterize pancreatic host immune responses during SFTSV infection, we performed a comprehensive transcriptomic analysis of pancreatic tissues from IFNAR^−/−^ mice. PCA analysis showed that SFTSV infection significantly altered the transcriptome of the mouse pancreas (Figure S6A, Supporting Information). Viral transcripts were exclusively detected in infected tissues and not in the control group (Figure S6B, Supporting Information). DEGs were identified using thresholds of |log_2_ fold‐change| ≥1 and adjusted *p*‐value < 0.05, yielding 1352 upregulated and 225 downregulated genes (Figure S6C, Supporting Information). KEGG enrichment analysis of upregulated genes revealed that the top 20 terms were all focused on immune responses, including proinflammatory pathways such as MAPK and JAK‐STAT signaling pathways (Figure 3E). Consistently, cytokine production and chemokine production such as *Ccl3, Ccl5, Tnf* and *Il1b* were significantly increased (Figure 3F, G). Moreover, cell death‐related genes such as *Ripk3, Aim2 and Nlrp3* were also upregulated in SFTSV infected pancreatic tissue (Figure S6D, Supporting Information), and immunofluorescence assay also confirmed that cell death occurred in the pancreatic tissue of SFTSV‐infected mice (Figure S6E,F, Supporting Information). Mirroring pancreatic organoid studies, pro‐pancreatitis genes were also upregulated in the pancreatic tissues of mice infected with SFTSV (Figure S6G,H, Supporting Information). At the same time, the protein expression level of Icam1 was also significantly increased after SFTSV infection (Figure S6I, Supporting Information). Taken together, these results demonstrated that direct SFTSV infection triggers intense pancreatic innate immune responses, characterized by cell death, cytokine storm, and immune infiltration, culminating in tissue damage.

### 2.4 Similarities and Differences in Host Responses to SFTSV and Cerulein‐Induced Pancreatitis

**Figure 4:**
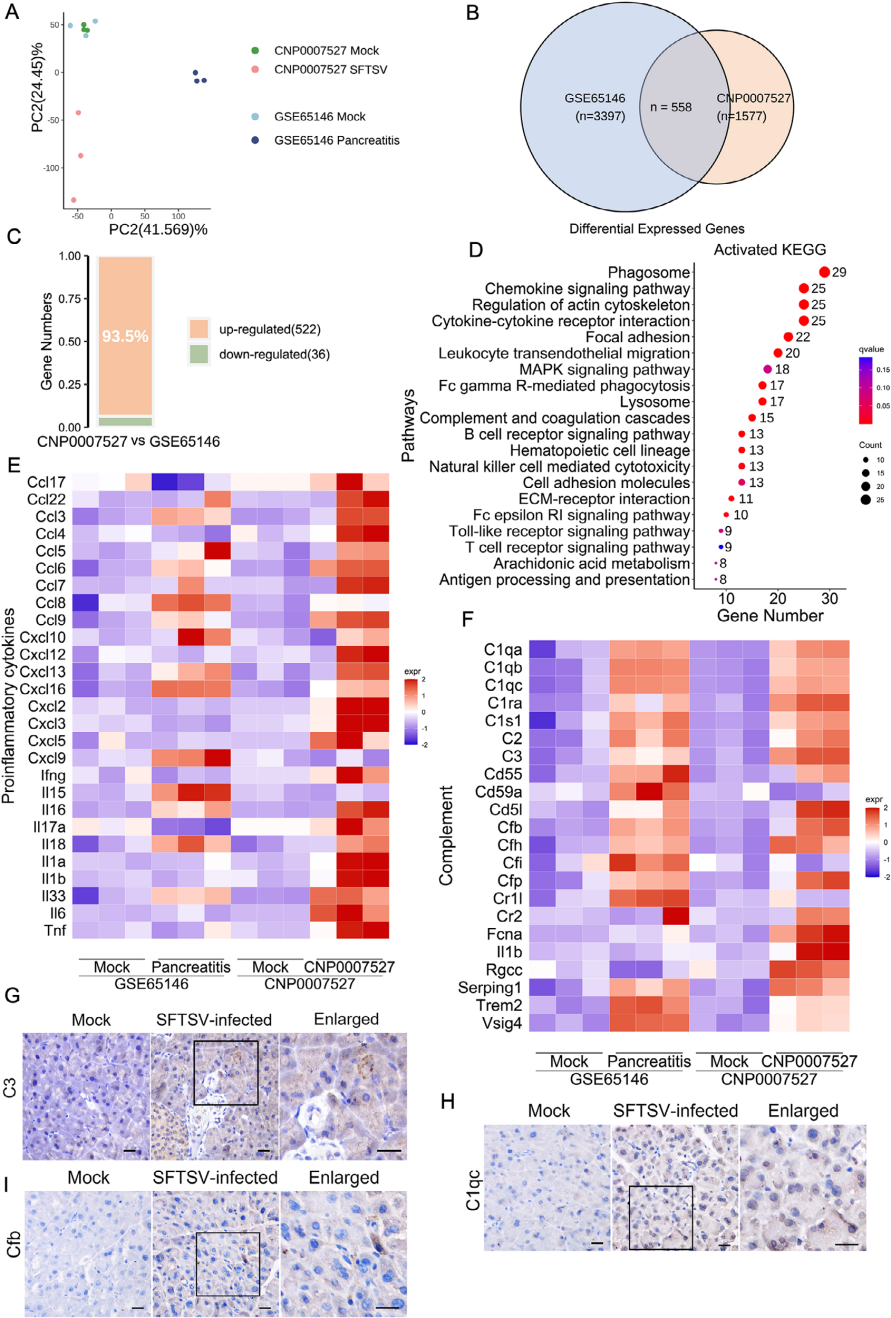
Transcriptome similarities and differences between viral pancreatitis and cerulein‐induced pancreatitis. A) PCA reveals transcriptomic differences between the viral infection and cerulein‐induced pancreatitis models, with three biological replicates per group. B) Venn diagram of DEGs between pancreatic tissues from cerulein‐induced and SFTSV infected mice, only DEGs with the same direction of change are counted as common DEGs. C) Stacked plot shows the direction and proportion of common DEGs in SFTSV infection and cerulein‐induced pancreatitis. D) The bubble plot shows the top 20 terms in KEGG analysis of upregulated genes of common DEGs in SFTSV infection and cerulein‐induced pancreatitis. E) The heatmap shows the representative proinflammatory cytokine‐related genes differential expression between SFTSV infection and cerulein‐induced pancreatitis among the common DEGs. F) The heatmap shows the representative complement genes among the common DEGs. G–I) Immunohistochemical detection of C3 G), C1qc H), and Cfb I) in murine pancreatic tissues. Scale bar, 20 µm.

To gain deeper insights into the pathogenesis of viral pancreatitis, we conducted a comparative transcriptomic analysis of pancreatic tissues from SFTSV‐infected mice (CNP0007527) versus cerulein‐induced acute pancreatitis (GSE65146). PCA revealed distinct transcriptomic profiles between the two models (**Figure** **4**A). Among 1577 DEGs in SFTSV infection and 3397 DEGs in cerulein‐induced pancreatitis, only 558 genes overlapped (Figure 4B). Notably, 522 (93.5%) of these shared DEGs were upregulated, with merely 36 downregulated (Figure 4C). KEGG analysis showed that the shared upregulated genes were mainly related to immune response, including MAPK signaling, complement and coagulation cascades, and cytokine‐chemokine production (Figure 4D), indicating immune hyperactivation as a common pathogenic feature. Crucially, SFTSV infection induced significantly higher expression of multiple cytokines and chemokines than cerulein treatment (Figure 4E), but this may also be related to the use of IFNAR^−/−^ mice. The upregulation of a series of complement‐related genes is particularly noteworthy (Figure 4F). The high protein expression of C1qc, C3, and Cfb was also confirmed through IHC (Figure 4G–I). The complement and coagulation cascade pathways were identified as common pathways in pancreatitis.^[^ ^29^ ^]^

## 3 Discussion

The alarming current situation of SFTS, characterized by rapidly increasing incidence, expanding prevalence areas, and persistently high mortality rates, is compounded by the absence of specific treatments or vaccines.^[^ ^7^ ^]^ To mitigate this public health crisis, it is crucial for us to understand the pathogenesis of SFTS. Here, we demonstrate the pancreatic tropism of SFTSV in both mouse models and ex vivo organoid models, with expression of viral entry receptors CCR2 ^[^ ^17^ ^]^ and LRP1 ^[^ ^18^ ^]^ in pancreatic tissue theoretically supporting this phenotype. SFTSV infection in pancreatic tissue provokes key immune events including cell death, complement activation, and inflammatory cascades, leading to tissue damage. More importantly, some patients with SFTS were indeed diagnosed with viral pancreatitis clinically. These current data suggest that pancreatic involvement is a previously underrecognized component of SFTS and warrants future studies to quantify its impact on disease progression.

Unexpectedly, our retrospective analysis of the SFTS cohort revealed not only elevated hepatic inflammation markers (ALT, AST) as previously documented, but also significantly increased amylase and lipase levels—indicators of pancreatitis. More interestingly, the levels of these two indicators were positively correlated with viral load. Combining clinical symptoms such as abdominal pain, 17.6% of patients were diagnosed with viral pancreatitis. Unlike other pancreatitis, viral pancreatitis does not present with typical symptoms on CT scans. Although pancreatic necrosis was observed on the abdominal CT of the SFTS patient in Tian et al.’s study,^[^ ^30^ ^]^ he did not observe similar phenomena in other SFTS patients with viral pancreatitis, and that patient ultimately died of multiple organ failure, which may be an isolated case and not representative. Viral pancreatitis can also occur in infections caused by many pathogens, such as Epstein‐Barr virus (EBV),^[^ ^31^, ^32^ ^]^ Coxsackievirus,^[^ ^33^ ^]^ SARS‐CoV‐2 ^[^ ^34^ ^]^ and so on, but the exact mechanism of its occurrence remains unclear.

Multiorgan damage is commonly reported in SFTSV infection, especially in severe cases.^[^ ^9^ ^]^ Mechanistically, it is mostly attributed to systemic inflammation, such as the virus inducing NETosis to downregulate specific inflammatory factors, hindering the initiation of immune responses and the process of organ repair, leading to liver and spleen damage.^[^ ^35^ ^]^ Humanized mouse model studies revealed that SFTSV infection increased vascular permeability by promoting the internalization of endothelial cell adhesion molecule VE‐cadherin, while the inflammatory factor storm caused by infection significantly aggravated vascular endothelial injury.^[^ ^36^ ^]^ Additionally, there has been controversy regarding the target organs and cells of SFTSV within the body. An autopsy study found that SFTSV infecting B‐lineage lymphocytes was a primary cause of systemic multi‐organ infection,^[^ ^12^ ^]^ whereas murine models show viral colocalization with splenic and hepatic macrophages, suggesting macrophages act like “Trojan horse”‐mediated virus spread.^[^ ^36^ ^]^ But here, both ex vivo organoid models and murine models have demonstrated that SFTSV can directly infect the pancreas. The intense inflammatory immune response caused by infection can also be visualized in the transcriptome, which has similar characteristics to cerulein‐induced pancreatitis.

Due to the diverse causes, the molecular networks regulating pancreatitis development are intricate. In cerulein‐induced pancreatitis models, ferroptosis plays a crucial role in its pathogenesis.^[^ ^20^, ^37^ ^]^ CTSB activates trypsinogen in acinar cells, causing acinar cell death, followed by a pro‐inflammatory response and leukocyte migration, exacerbating local injury and increasing the severity of pancreatitis.^[^ ^19^ ^]^ Strikingly, the transcriptomes of pancreatic from mice infected with SFTSV and from mice treated with cerulein both highlight the central role of complement hyperactivation‐inflammation cascades in pancreatitis. Complement activation accelerates the severe progression of certain viral infections, and targeted complement therapy has emerged as a promising treatment strategy.^[^ ^38^, ^39^, ^40^ ^]^ C3 inhibitor AMY‐101 showed promising efficacy in clinical trials for severe COVID‐19,^[^ ^41^, ^42^ ^]^ but its effectiveness in SFTS treatment requires further study.

Given the complex role of type I interferons in immune responses to viral infections, using IFNAR^−/−^ mice to study immune responses induced by viral infections is not entirely appropriate. But the optional SFTS mouse model is highly limited, as wild‐type mice infected with SFTSV experience self‐limiting infection, with the virus being rapidly cleared and no tissue damage observed. Loss of interferon receptors promotes viral proliferation and may amplify inflammatory signals. However, strong interferon response and inflammatory factor storm are important immune features in patients with deceased SFTS,^[^ ^43^ ^]^ and interferon has a pro‐inflammatory effect in the later stage of SFTS development. In addition to observing SFTSV infection activating inflammatory responses in mouse pancreatic tissue, more importantly, SFTSV infection of pancreatic organoids also triggers a storm of interferon responses and inflammatory cytokines.

In summary, our results described above demonstrate that SFTSV can directly infect pancreatic tissue, triggering a series of innate immune responses that cause tissue damage. This is clinically evidenced by elevated amylase and lipase levels of SFTS patients, with 17.6% developing abdominal pain‐compatible viral pancreatitis. Omics studies on in vivo and in vitro infection models have revealed the crucial regulatory role of the complement‐inflammatory cascade, suggesting that this may be a therapeutic target for SFTS.

## 4 Experimental Section

### Ethics Statement

This work was conducted following the guidelines of the Declaration of Helsinki of the World Medical Association and approved by the Ethics Committee of the Second Hospital of Anhui Medical University (YX2025‐035). All patients had been informed prior to hospitalization that their clinical data or samples would be used for scientific research, and they signed informed consent. All patient data were anonymized and de‐identified prior to analysis.

### Patient Recruitment

The clinical information, including viral load, biochemical tests, and immunological indicators, of SFTS patients from the First and Second Affiliated Hospitals of Anhui Medical University over the past five years was collected. Patients with incomplete clinical data were excluded, resulting in a cohort of 290 patients for analysis. The patients were confirmed of SFTSV infection by qRT‐PCR. Viral Pancreatitis diagnosis was defined according to the revision of the Atlanta criteria.^[^ ^13^ ^]^ Other etiologies for pancreatitis, such as cholelithiasis, alcoholism, drugs, other types of infectious pancreatitis, hypercalcemia, hyperlipidemia, and autoimmune conditions, were excluded.

### Human Pancreatic Organoid Culture

The Medical Ethics Committee of the Second Hospital of Anhui Medical University approved the research (S20210059). Fresh samples were washed with transport media, mechanical dissection into small pieces, enzymatic digestion in a volume with media I (DMEM (Gibco), 100 U mL^−1^ penicillin, 100 mg mL^−1^ streptomycin (Gibco), 250 U mL^−1^ collagenase IV (Life Technologies), 100 mg mL^−1^ Primocin (InvivoGen), and 10 mmol L^−1^ Rock inhibitor Y‐27632) and incubated on a shaker at 200 rpm at 37 °C. The pellet pieces were digested again with media II (DMEM (Gibco), 100 U mL^−1^ penicillin, 100 mg mL^−1^ streptomycin (Gibco), 0.05%vTrypsin‐EDTA(Gibco), 100 mg mL^−1^ Primocin (InvivoGen), and 10 mmol L^−1^ Rock inhibitor Y‐27632) for ≈5–10 min. The supernatants from both digestion steps were pooled, and FBS was added to inactivate the enzymes. Suspension was centrifuged at 300 g for 5 min, and the cell pellet was washed once with washing media. The cells were resuspended in a small volume of tissue‐type‐specific culture media, using Matrigel to dilute cell suspension with Matrigel: Cell = 2:1, and 100 µL Matrigel/cell mixture was distributed into a 6‐well cell suspension culture plate. Invert the culture plate, and the drops were allowed to polymerize for 30 min inside the incubator at 37 °C and 5% CO2. 3 mL tumor‐type–specific culture media were added per well. Refresh the culture medium every three days during organoid culture.

### SFTSV Preparation and Infection

The SFTSV (AH001) used in the experiments was propagated in Vero E6 cells, and the viral titer was determined using a plaque‐forming assay on Vero cells. A titer of 10^7^ PFU mL^−1^ virus was used as a stock. Human pancreatic organoids were incubated with SFTSV at a multiplicity of infection (MOI) of 1 for 2 h at 37 °C. Subsequently, the infection medium was removed and cells were cultured at 37 °C and 5% CO2 in DMEM supplemented with 2% fetal bovine serum (FBS) and 1% penicillin–streptomycin.

### Evaluate the Impact of SFTSV Infection on Mouse Pancreas

The Animal Ethics Committee of the Second Hospital of Anhui Medical University approved the research (LLSC20200982). The animal experiments were conducted in the BLS‐3 animal facility approved for the studies of SFTSV. 8‐week‐old IFNAR^−/‐^ C57BL/6 mice were intraperitoneally inoculated with SFTSV at 10^4^ PFU per mouse. Independent experiments were performed to collect tissue samples. A subset of mice was marked at the beginning and the mice were euthanized at 4 dpi. Pancreas samples were collected for further analysis.

### qRT‐PCR

Human pancreatic organoids were infected with SFTSV at a MOI of 1 or mock‐treated. At 48 hpi, organoids were harvested and total RNA was isolated using RNAiso Plus Reagent (9109, Takara). IFNAR^−/−^ mice were infected with 10^4^ PFU SFTSV or control‐treated for 4 days and pancreatic tissues were harvested. Total RNA was extracted using RNAiso Plus Reagent (9109, Takara) according to the manufacturer's protocol. qRT‐PCR was performed with a two‐step procedure using the PrimeScript RT Master Mix (RR036A, Takara) and TB Green Premix Ex Taq II (Tli RNaseH Plus) (RR820A, Takara), and was analyzed on Roche LightCycler 96 SW 1.1 System (Roche). All results were standardized for GAPDH internal reference expression and calculated using 2^−ΔΔ^Ct method. Primer sequences were shown in Table S2 (Supporting Information).

### RNA‐Seq Library Construction

RNA purity and quantification were evaluated using the NanoDrop 2000 spectrophotometer (Thermo Scientific). RNA integrity was assessed using the Agilent 2100 Bioanalyzer (Agilent Technologies, Santa Clara, CA). Then the libraries were constructed using VAHTS Universal V6 RNA‐seq Library Prep Kit (NR604‐01, Vazyme) according to the manufacturer's instructions. The libraries were sequenced by OE Biotech Co., Ltd. (Shanghai, China) on an illumina Novaseq 6000 platform and 150 bp paired‐end reads were generated.

### RNA‐Seq Data Analysis

The quality of sequencing was assessed with FastQC (v0.11.8). The clean reads were obtained by using SOAPnuke (v1.3.0) to filter the low‐quality data, and aligned to the reference genome by STAR (v2.7.0) with default parameters. The processed reads from each sample were aligned with STAR against the reference sequence. The gene expression analyses were performed with Htseq (v0.11.2). DESeq (v1.28.0) was used to analyze the DEGs between samples. Differential gene expression analysis was based on the DESeq2. The *p*‐value was corrected by the FDR method to obtain the *q* value, which was used to conduct significance analysis. A combat algorithm from package sva (v3.21) was used to correct batch effects between datasets. Parameters for classifying significant DEGs are 2‐fold differences (|log2FC| ≥ 1, FC, the fold change of expressions) in the transcript abundance and *p* < 0.05). To explore the biological functions of these differentially expressed genes, we performed functional enrichment analysis using cluster Profiler, including KEGG pathway analysis. For data visualization, it was utilized ggplot2 to create statistical graphics such as volcano plots, and the Complexheatmap package to generate heatmaps that intuitively display the expression patterns of differentially expressed genes across various samples.

### Quantification of SFTSV Genomes

Raw paired‐end sequencing reads were first processed using Trim Galore (v0.6.7), low‐quality bases with a Phred score below 20 were trimmed from the ends of reads, and any reads shorter than 35 base pairs after trimming were discarded. Adapter sequences were also removed with a stringency setting of 3. Then the data underwent rRNA depletion. The quality‐filtered reads were aligned to an rRNA reference database using the Bowtie aligner. Critically, only the unmapped read pairs, representing the non‐rRNA fraction of the transcriptome, were retained for subsequent analysis. The SFTSV(AH001) sequence was used as the reference genome, and an index was built with STAR for viral genome quantification. These cleaned, rRNA‐depleted reads were then aligned to the reference genome using the STAR aligner. The resulting alignments were directly output as a coordinate‐sorted BAM file indexed using Samtools (v1.17) to enable efficient access for SFTSV genome quantification.

### Western Blot Analysis

Cells were collected and lysed with the RIPA lysis buffer (P0013B, Beyotime). Pancreatic tissue is homogenized by grinding with RIPA lysis buffer. Proteins were separated by SDS–PAGE and transferred onto a polyvinylidene difluoride (PVDF) membrane. All the membranes were blocked with 5% (w/v) nonfat dry milk in TBST for 1 h at room temperature. Proteins were incubated with the indicated primary antibodies including rabbit GAPDH polyclonal antibody (10494‐1‐AP, Proteintech) and mouse anti‐SFTSV NP (self‐prepared). After incubation with the primary antibody overnight at 4 °C, the membranes were washed with TBST and then incubated for 1 h at room temperature with the corresponding HRP‐conjugated Goat Anti‐Rabbit IgG(H+L) (SA00001‐2, Proteintech) or HRP‐conjugated Goat Anti‐Mouse IgG(H+L) (SA00001‐1, Proteintech).

### H&E Staining and Immunohistochemical Staining

The sections of human pancreatic organoids and pancreatic tissues were fixed with 4% paraformaldehyde, embedded in paraffin and cut into sections of 4 µm, and further used for histology or immunostaining. For histology, sections were stained with Gill's hematoxylin and eosin‐Y. For immunohistochemical staining, the slices were dewaxed and subjected to antigen retrieval by immersion in citrate buffer (pH 6.0). After three washes with PBS, endogenous peroxidase activity was blocked with an endogenous peroxidase blocker (P0100B, Beyotime) to reduce background interference. The sections were subsequently washed three times with PBS and blocked with 5% BSA for 1 h at room temperature to prevent nonspecific binding. The primary antibodies including mouse anti‐SFTSV NP, rabbit anti CK19(10712‐1‐AP, Proteintech), rabbit anti amylase (3796, Cell Signaling Technology) rabbit anti‐CD68 (28058‐1‐AP, Proteintech), and rabbit anti‐Ly‐6G (YM8307, Immunoway) were applied, and incubated overnight at 4 °C. The slides were returned to room temperature for 40 min, washed three times with PBS, and then incubated with an appropriate secondary antibody including HRP‐conjugated Goat Anti‐Mouse IgG (D110087, Sangon Biotech) or Goat anti‐Rabbit IgG (31 460, Invitrogen) for 1 h at room temperature. Following three additional PBS washes, a DAB substrate was used for color development for 30–60 s, which resulted in a brown precipitate. The sections were counterstained with hematoxylin for 1 min, blueing was achieved with lithium carbonate, and finally, the slides were cleared and mounted with coverslips.

### Immunofluorescence Assay

The sections of SFTSV‐infected human pancreatic organoids were dewaxed and subjected to antigen retrieval by immersion in citrate buffer (pH 6.0), heated in a microwave for 10–15 min until boiling, and then allowed to cool to room temperature. Pancreas samples were embedded with OCT (Biosharp) and sectioned into 8 µm thin slices with a microtome, and rewarmed to room temperature. Monolayer cells were fixed with 4% (w/v) paraformaldehyde for 30 min. After three washes with PBS, the sections and cells were then permeabilized with 0.01% Triton X‐100 for 10 min, and then blocked with 5% (w/v) bovine serum albumin (BSA) for 1 h at room temperature. To detect the localization of SFTSV, mouse anti‐SFTSV NP was used as primary antibody. For the co‐staining of NP and Cleaved Caspase‐3 in the pancreas of IFNAR^−/−^ mice, mouse anti‐SFTSV NP and rabbit anti‐Cleaved Caspase‐3 (9664, Cell Signaling Technology) were used as primary antibodies overnight at 4 °C. The sections and cells were then incubated with the appropriate antibodies, goat anti‐mouse IgG 488 (A11029, Invitrogen) for mouse anti‐SFTSV NP and goat anti‐rabbit IgG 594 (A‐11012, Invitrogen) for rabbit anti‐Cleaved Caspase‐3 for 1 h at room temperature, and then washed and incubated with DAPI (C1006, Beyotime), and analyzed using a confocal microscope (LSM980, Zeiss).

### TUNEL Assay

TUNEL assay was performed using a One‐step TUNEL Apoptosis Assay Kit (KTA2011, Abbkine) according to the manufacturer's instructions. Immunostaining was performed using indicated primary antibodies (mouse anti‐SFTSV NP) and followed by incubation with Fluor 488 secondary antibody and DAPI, and analyzed using a confocal microscope.

### Statistical Analysis

All the experiments were replicated and the number of replicates was listed in the figure legends. For categorical variables, hypothesis testing employed Chi‐squared or Fisher's exact tests based on expected frequencies. Continuous variables were analyzed using Student's t‐test (normally distributed data) or Mann‐Whitney U test (non‐parametric data), with distribution normality assessed by Shapiro‐Wilk testing. All statistical tests used α = 0.05 significance threshold, with False Discovery Rate (FDR) correction for multiple comparisons. Principal Component Analysis (PCA) generated low‐dimensional data representations using the first two principal components. Linear relationships between variables were quantified via Pearson correlation coefficients. Analyses and visualizations were performed in R (v4.4.2). QRT‐PCR data were analyzed using GraphPad Prism 8.0 (GraphPad Software, CA). Statistical significance was determined by Student's t‐tests (two‐tailed) for two groups, or one‐way ANOVA for three or more groups, with a *p*‐value less than 0.05 considered statistically significant. Data were derived from the average of three biological replicate experiments, and calculated as the means ± SEM.

The data that support the findings of this study have been deposited into CNGB Sequence Archive (CNSA) with accession numbers CNP0007527 (Mouse pancreas), and CNP0007528 (Pancreatic organoids). The raw·NGS data of the SFTSV (AH001) can be accessed through project PRJNA1190154 as previously described.^[^ ^18^ ^]^

## Conflict of Interest

The authors declare no conflict of interest.

## Author Contributions

X.L., Z.X., Y.T., and C.W. contributed equally to this work. This project was designed and directed by G.X. X.L. in G.X. lab conducted SFTSV infection experiments. Z.X. in Y.D. lab performed the bioinformatics analysis. Y.T. generated human pancreatic organoids. Z.Z. is responsible for the clinical diagnosis of patients. Y.D. and Z.Z. collected the patients’ clinical information. Y.Y., C.W., J.M. and S.Z. contributed some experimental data. The manuscript was written by X.L. and G.X. and revised by Y.D., Z.Z. and G.X.

## Supporting information



Supporting Information

## Data Availability

The data that support the findings of this study are available from the corresponding author upon reasonable request.

## References

[1] X.‐J. Yu , M.‐F. Liang , S.‐Y. Zhang , Y. Liu , J.‐D. Li , Y.‐L. Sun , L. Zhang , Q.‐F. Zhang , V. L. Popov , C. Li , J. Qu , Q. Li , Y.‐P. Zhang , R. Hai , W. Wu , Q. Wang , F.‐X. Zhan , X.‐J. Wang , B. Kan , S.‐W. Wang , K.‐L. Wan , H.‐Q. Jing , J.‐X. Lu , W.‐W. Yin , H. Zhou , X.‐H. Guan , J.‐F. Liu , Z.‐Q. Bi , G.‐H. Liu , J. Ren , et al., N. Engl. J. Med. 2011, 364, 1523. 21410387 10.1056/NEJMoa1010095 PMC3113718

[2] A. M. Win , Y. T. H. Nguyen , Y. Kim , N.‐Y. Ha , J.‐G. Kang , H. Kim , B. San , O. Kyaw , W. W. Htike , D.‐O. Choi , K.‐H. Lee , N.‐H. Cho , Emerg Infect Dis 2020, 26, 1878. 32687023 10.3201/eid2608.200135 PMC7392420

[3] X. C. Tran , Y. Yun , L. Van An , S.‐H. Kim , N. T. P. Thao , P. K. C. Man , J. R. Yoo , S. T. Heo , N.‐H. Cho , K. H. Lee , Emerg. Infect. Dis. 2019, 25, 1029. 31002059 10.3201/eid2505.181463 PMC6478219

[4] Y. R. Kim , Y. Yun , S. G. Bae , D. Park , S. Kim , J. M. Lee , N.‐H. Cho , Y. S. Kim , K. H. Lee , Emerg. Infect. Dis. 2018, 24, 2103. 30334706 10.3201/eid2411.170756 PMC6199997

[5] P. Rattanakomol , S. Khongwichit , P. Linsuwanon , K. H. Lee , S. Vongpunsawad , Y. Poovorawan , Emerg. Infect. Dis. 2022, 28, 2572. 36418010 10.3201/eid2812.221183 PMC9707585

[6] D. Miao , M.‐J. Liu , Y.‐X. Wang , X. Ren , Q.‐B. Lu , G.‐P. Zhao , K. Dai , X.‐L. Li , H. Li , X.‐A. Zhang , W.‐Q. Shi , L.‐P. Wang , Y. Yang , L.‐Q. Fang , W. Liu , Clin. Infect. Dis. 2021, 73, 3851.

[7] H. Cui , S. Shen , L. Chen , Z. Fan , Q. Wen , Y. Xing , Z. Wang , J. Zhang , J. Chen , B. La , Y. Fang , Z. Yang , S. Yang , X. Yan , S. Pei , T. Li , X. Cui , Z. Jia , W. Cao , Lancet Reg. Health ‐ West Pac. 2024, 48, 101133. 39040038 10.1016/j.lanwpc.2024.101133 PMC11261768

[8] Annual Review of Diseases Prioritized under the Research and Development Blueprint, World Health Organization 2017.

[9] Z.‐T. Gai , Y. Zhang , M.‐F. Liang , C. Jin , S. Zhang , C.‐B. Zhu , C. Li , X.‐Y. Li , Q.‐F. Zhang , P.‐F. Bian , L.‐H. Zhang , B. Wang , N. Zhou , J.‐X. Liu , X.‐G. Song , A. Xu , Z.‐Q. Bi , S.‐J. Chen , D.‐X. Li , J. Infect. Dis. 2012, 206, 1095. 22850122 10.1093/infdis/jis472

[10] M. Saijo , J. Infect. Chemother. 2018, 24, 773. 30098914 10.1016/j.jiac.2018.07.009

[11] H. Li , Q.‐B. Lu , B. Xing , S.‐F. Zhang , K. Liu , J. Du , X.‐K. Li , N. Cui , Z.‐D. Yang , L.‐Y. Wang , J.‐G. Hu , W.‐C. Cao , W. Liu , Lancet Infect. Dis. 2018, 18, 1127. 30054190 10.1016/S1473-3099(18)30293-7

[12] T. Suzuki , Y. Sato , K. Sano , T. Arashiro , H. Katano , N. Nakajima , M. Shimojima , M. Kataoka , K. Takahashi , Y. Wada , S. Morikawa , S. Fukushi , T. Yoshikawa , M. Saijo , H. Hasegawa , J. Clin. Invest. 2020, 130, 799. 31904586 10.1172/JCI129171 PMC6994144

[13] P. A. Banks , T. L. Bollen , C. Dervenis , H. G. Gooszen , C. D. Johnson , M. G. Sarr , G. G. Tsiotos , S. S. Vege , Gut 2013, 62, 102. 23632143 10.1016/j.suc.2013.02.012

[14] T. J. Harris , W. C. Beck , A. Bhavaraju , B. Davis , M. K. Kimbrough , J. C. Jensen , A. Privratsky , J. R. Taylor , K. W. Sexton , J. Surg. Case Rep. 2018, 2018, rjy048. 29644032 10.1093/jscr/rjy048 PMC5887590

[15] J. T. Kleinman , R. K. Sivamani , V. M. Kelly , Gastroenterology 2010, 138, 7. 10.1053/j.gastro.2009.09.076 20347040

[16] S. F. Boj , C.‐I. Hwang , L. A. Baker , I. I. C. Chio , D. D. Engle , V. Corbo , M. Jager , M. Ponz‐Sarvise , H. Tiriac , M. S. Spector , A. Gracanin , T. Oni , K. H. Yu , R. van Boxtel , M. Huch , K. D. Rivera , J. P. Wilson , M. E. Feigin , D. Öhlund , A. Handly‐Santana , C. M. Ardito‐Abraham , M. Ludwig , E. Elyada , B. Alagesan , G. Biffi , G. N. Yordanov , B. Delcuze , B. Creighton , K. Wright , Y. Park , et al., Cell 2015, 160, 324. 25557080 10.1016/j.cell.2014.12.021 PMC4334572

[17] L. Zhang , X. Peng , Q. Wang , J. Li , S. Lv , S. Han , L. Zhang , H. Ding , C.‐Y. Wang , G. Xiao , X. Du , K. Peng , H. Li , W. Liu , Sci. Adv. 2023, 9, adg6856. 10.1126/sciadv.adg6856 PMC10396298 37531422

[18] C. Xing , C. Zhang , Z. Xu , Y. Wang , W. Lu , X. Liu , Y. Zhang , J. Ma , S. Yang , Y. Du , G. Xu , Y. Liu , Nat. Commun. 2025, 16, 4036. 40301361 10.1038/s41467-025-59305-0 PMC12041282

[19] M. Sendler , F.‐U. Weiss , J. Golchert , G. Homuth , C. van den Brandt , U. M. Mahajan , L.‐I. Partecke , P. Döring , I. Gukovsky , A. S. Gukovskaya , P. R. Wagh , M. M. Lerch , J. Mayerle , Gastroenterology 2018, 154, 704. 29079517 10.1053/j.gastro.2017.10.018 PMC6663074

[20] L. Yang , F. Ye , J. Liu , D. J. Klionsky , D. Tang , R. Kang , Autophagy, 19, 1733. 10.1080/15548627.2022.2152209 PMC10262765 36426912

[21] R. Seelig , H. P. Seelig , Z. Gastroenterol. 1976, 14, 654. 136818

[22] R. Seelig , H. P. Seelig , Virchows Arch. Pathol. Anat. Histol. 1976, 371, 69. 10.1007/BF00433716 822574

[23] J. K. Horn , J. H. Ranson , I. M. Goldstein , J. Weissler , D. Ceratola , R. Taylor , H. D. Perez , Am. J. Pathol. 1980, 101, 205. 6160768 PMC1903594

[24] I. M. Goldstein , D. Cala , A. Radin , H. B. Kaplan , J. Horn , J. Ranson , Am. J. Med. Sci. 1978, 275, 257. 80133 10.1097/00000441-197805000-00003

[25] X. Wang , Z. Sun , A. Börjesson , R. Andersson , J. Br. Surg. 1999, 86, 411. 10.1046/j.1365-2168.1999.01028.x 10201790

[26] M. Sendler , A. Dummer , F. U. Weiss , B. Krüger , T. Wartmann , K. Scharffetter‐Kochanek , N. van Rooijen , S. R. Malla , A. Aghdassi , W. Halangk , M. M. Lerch , J. Mayerle , Gut 2013, 62, 430. 22490516 10.1136/gutjnl-2011-300771

[27] H. Sternby , H. Hartman , H. Thorlacius , S. Regnér , Biomolecules 2021, 11, 591. 33920566 10.3390/biom11040591 PMC8073083

[28] P. Kaufmann , K. H. Smolle , G. A. Brunner , U. Demel , G. P. Tilz , G. J. Krejs , Am. J. Gastroenterol. 1999, 94, 2412. 10484001 10.1111/j.1572-0241.1999.01366.x

[29] Q. Liu , L. Li , D. Xu , J. Zhu , Z. Huang , J. Yang , S. Cheng , Y. Gu , L. Zheng , X. Zhang , H. Shen , Front. Cell. Infect. Microbiol. 2022, 12, 1052466. 36590588 10.3389/fcimb.2022.1052466 PMC9795030

[30] B. Tian , D. Qu , A. Sasaki , J. Chen , B. Deng , Pancreatology 2020, 20, 1631. 33092955 10.1016/j.pan.2020.09.024

[31] S.‐J. Kang , K.‐H. Yoon , J.‐B. Hwang , Pediatr. Gastroenterol. Hepatol. Nutr. 2013, 16, 61. 24010108 10.5223/pghn.2013.16.1.61 PMC3746049

[32] S. Singh , P. Khosla , J. Infect. Public Health 2016, 9, 98. 26190854 10.1016/j.jiph.2015.06.011

[33] C. Vella , C. L. Brown , D. A. McCarthy , J. Gen. Virol. 1992, 73, 1387. 1607860 10.1099/0022-1317-73-6-1387

[34] Y. Miao , O. Lidove , W. Mauhin , Br. J. Surg. 2020, 107, 270. 10.1002/bjs.11741 PMC7300914 32492174

[35] X. Ji , X. Yu , Z. Xiao , R. Zhang , Z. Wu , X. Zhang , C. Wang , J. Zhu , Y. Yang , T. Zhou , J. Leukocyte Biol. 2025, 117, qiaf053. 40275780 10.1093/jleuko/qiaf053

[36] S. Xu , N. Jiang , W. Nawaz , B. Liu , F. Zhang , Y. Liu , X. Wu , Z. Wu , PLoS Pathog. 2021, 17, 1009587. 10.1371/journal.ppat.1009587 PMC8139491 33974679

[37] Y. Liu , H. Cui , C. Mei , M. Cui , Q. He , Q. Wang , D. Li , Y. Song , J. Li , S. Chen , C. Zhu , Cell Death Dis. 2023, 14, 694. 37865653 10.1038/s41419-023-06216-x PMC10590376

[38] L. Ma , S. K. Sahu , M. Cano , V. Kuppuswamy , J. Bajwa , J.’N. McPhatter , A. Pine , M. L. Meizlish , G. Goshua , C. H. Chang , H. Zhang , C. Price , P. Bahel , H. Rinder , T. Lei , A. Day , D. Reynolds , X. Wu , R. Schriefer , A. M. Rauseo , C. W. Goss , J. A. O'Halloran , R. M. Presti , A. H. Kim , A. E. Gelman , C. S. Dela Cruz , A. I. Lee , P. A. Mudd , H. J. Chun , J. P. Atkinson , et al., Sci. Immunol. 2021, 6, abh2259. 10.1126/sciimmunol.abh2259 PMC8158979 34446527

[39] P. Georg , R. Astaburuaga‐García , L. Bonaguro , S. Brumhard , L. Michalick , L. J. Lippert , T. Kostevc , C. Gäbel , M. Schneider , M. Streitz , V. Demichev , I. Gemünd , M. Barone , P. Tober‐Lau , E. T. Helbig , D. Hillus , L. Petrov , J. Stein , H.‐P. Dey , D. Paclik , C. Iwert , M. Mülleder , S. K. Aulakh , S. Djudjaj , R. D. Bülow , H. E. Mei , A. R. Schulz , A. Thiel , S. Hippenstiel , A.‐E. Saliba , et al., Cell 2022, 185, 493. 35032429 10.1016/j.cell.2021.12.040 PMC8712270

[40] J. Nitkiewicz , A. Borjabad , S. Morgello , J. Murray , W. Chao , L. Emdad , P. B. Fisher , M. J. Potash , D. J. Volsky , J. Neuroinflammation 2017, 14, 23. 28122624 10.1186/s12974-017-0794-9 PMC5267445

[41] S. Mastaglio , A. Ruggeri , A. M. Risitano , P. Angelillo , D. Yancopoulou , D. C. Mastellos , M. Huber‐Lang , S. Piemontese , A. Assanelli , C. Garlanda , J. D. Lambris , F. Ciceri , Clin. Immunol. 2020, 215, 108450. 32360516 10.1016/j.clim.2020.108450 PMC7189192

[42] P. Skendros , G. Germanidis , D. C. Mastellos , C. Antoniadou , E. Gavriilidis , G. Kalopitas , A. Samakidou , A. Liontos , A. Chrysanthopoulou , M. Ntinopoulou , D. Kogias , I. Karanika , A. Smyrlis , D. Cepaityte , I. Fotiadou , N. Zioga , I. Mitroulis , N. K. Gatselis , C. Papagoras , S. Metallidis , H. Milionis , G. N. Dalekos , L. Willems , B. Persson , V. A. Manivel , B. Nilsson , E. S. Connolly , S. Iacobelli , V. Papadopoulos , R. T. Calado , et al., Sci. Adv. 2022, 8, abo2341. 10.1126/sciadv.abo2341 PMC9385148 35977025

[43] H. Li , X. Li , S. Lv , X. Peng , N. Cui , T. Yang , Z. Yang , C. Yuan , Y. Yuan , J. Yao , Z. Yuan , J. Li , X. Ye , X. Zhang , S. Zhu , K. Peng , W. Liu , Cell Rep. 2021, 37, 110039. 34818556 10.1016/j.celrep.2021.110039

