# Supplementary material for: Investigation into the Pancreatic Pathogenesis of SFTSV across Multiple Levels
Source: Adv Sci (Weinh). 2025 Oct 15;13(1):e15862. doi: 10.1002/advs.202515862 (PMC12767103; doi:10.1002/advs.202515862)

**Supplementary Materials**

**Investigation into the Pancreatic Pathogenesis of SFTSV across Multiple Levels**

Xiaohan Liu^1^, Zhihao Xu^2^, Yilun Tong^3^, Changtai Wang^4^, Yueqi Yao^1^, Yujie Diao ^5^, Jingyuan Ma^1^, Shijun Zhou^4^, Yinan Du^1^, Zhenhua Zhang^4^, Gang Xu^1,4^

^🖂^Correspondence: Yinan Du (duyinan@ahmu.edu.cn), Zhenhua Zhang (zhangzhenhua@ahmu.edu.cn), Gang Xu (xugang@ahmu.edu.cn)

**The Word file includes:**

Figures. S1-S6

Figure legends for Figs. S1-S6

Tables S1 to S2


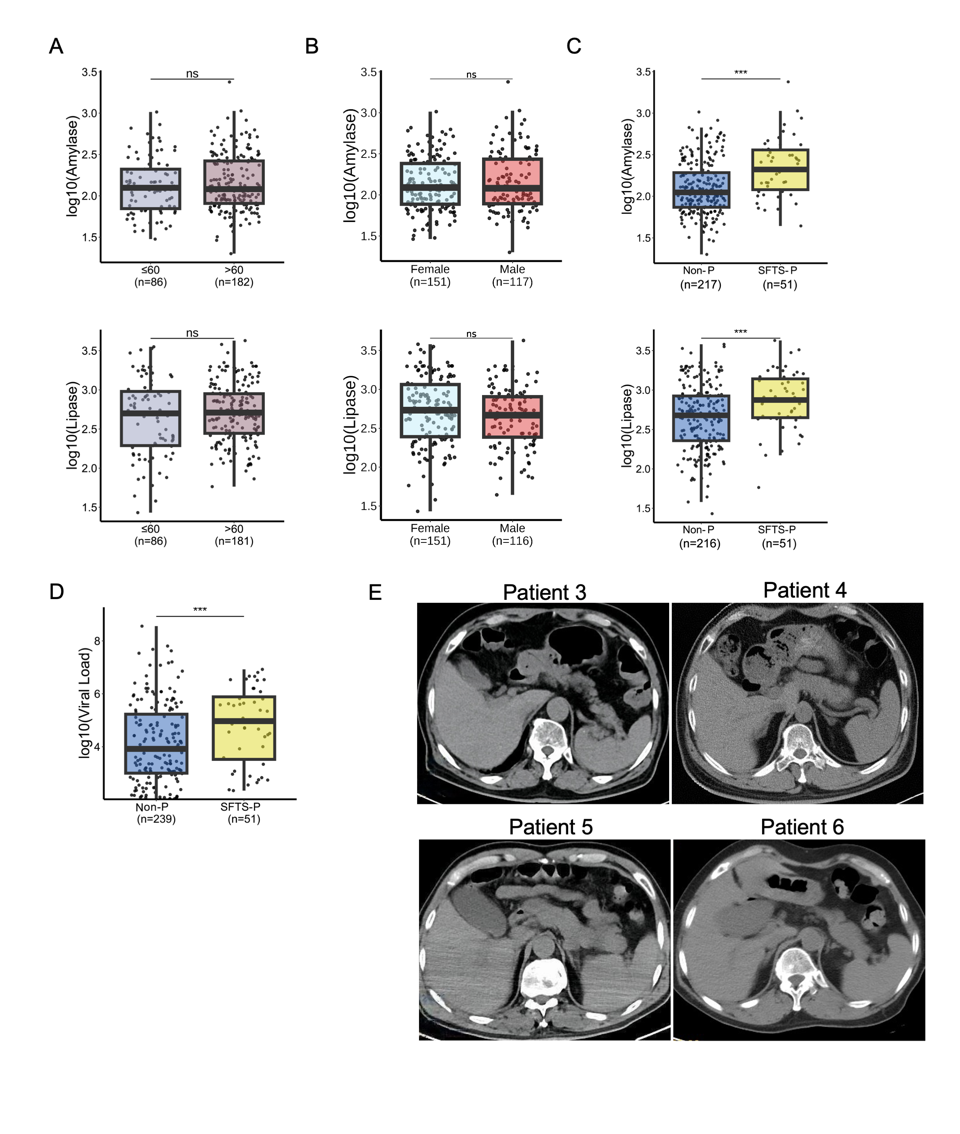


Figure S1 Serum amylase and lipase levels are significantly correlated with viral load

Boxplots illustrating the distribution of amylase (A) and lipase (B) in different age groups and sex groups of SFTS patients. Boxplot showing the amylase and lipase concentration (C), and viral load (D) distribution between SFTS patients without pancreatitis (Non-P) and SFTS patients with pancreatitis (SFTSV-P). (E) CT images of SFTS patients. Statistical significance was analyzed by Student’s t-test. *P < 0.05; **P < 0.01; ***P < 0.001; **** P < 0.0001; ns, no significance.


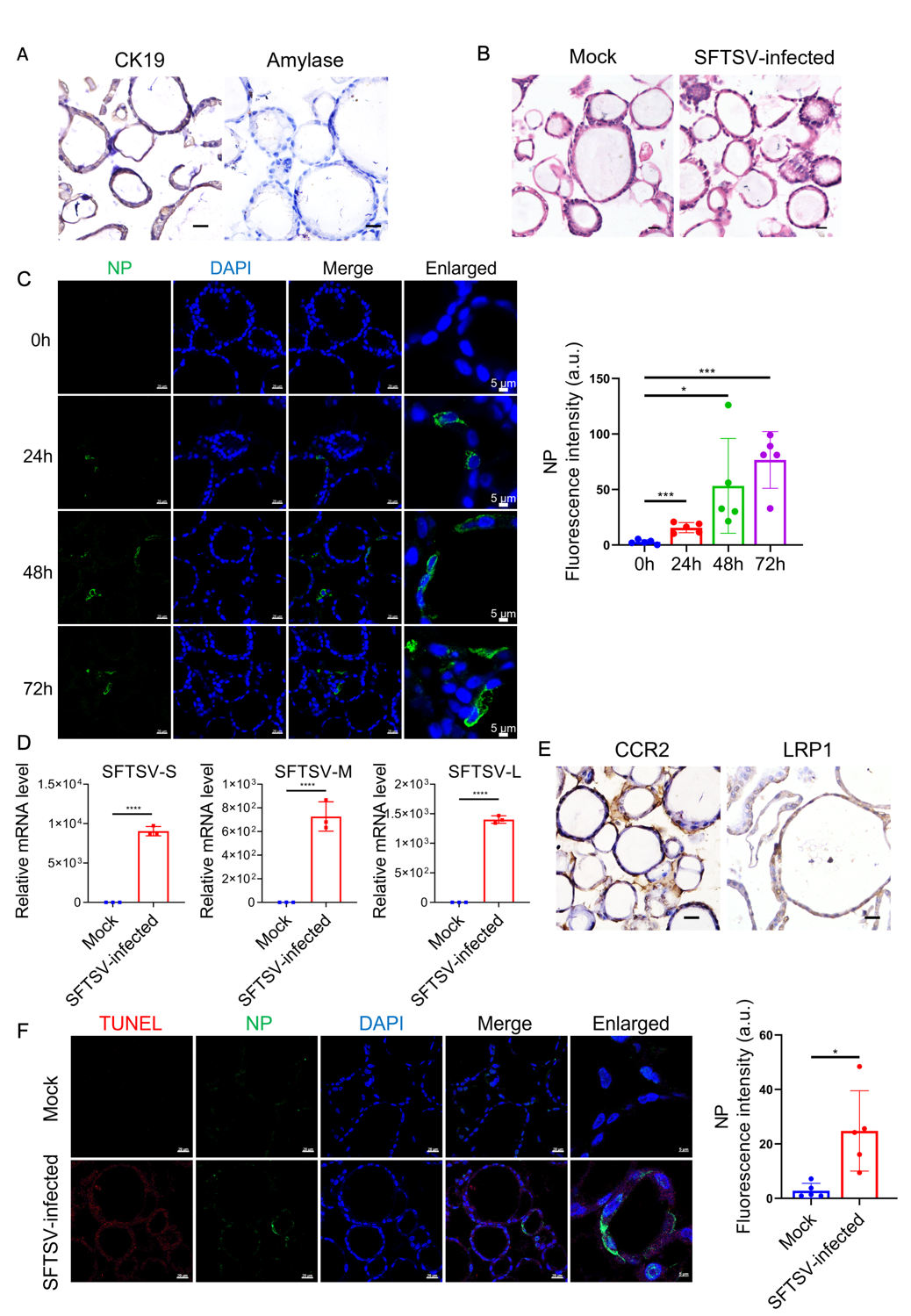


Figure S2 Direct infection of pancreatic organoids by SFTSV triggers cell death

(A) Immunohistochemical detection of CK19 and amylase expression in human pancreatic organoids. scale bars, 20 μm. (B) H&E staining of human pancreatic organoid sections showing structures. Scale bar, 20 μm. (C) Immunofluorescence detection of viral capsid protein NP expression (green) in human pancreatic organoids at different time points following mock and SFTSV infection. Scale bars, 20 μm and 5 μm in the enlarged image. Fluorescence intensity (arbitrary units, a.u.) of NP signals is shown on the right. Five fields of each section were quantified. (D) Relative mRNA expression levels of SFTSV genomic segments (S, M, L) by qRT-PCR. (E) Immunohistochemical detection of SFTSV receptors CCR2 and LRP1 expressions in human pancreatic organoids. Scale bar, 20 μm. (F) Immunofluorescence detection of TUNEL^+^ apoptotic cells (red) and NP (green) in pancreatic organoids with DAPI (blue). Scale bars, 20 μm and 5 μm in the enlarged image. Quantification is shown on the right. Five fields of each section were quantified. Data shown are means ± SEM. Statistical significance was analyzed by Student’s t-test. **P* < 0.05; ***P* < 0.01; ****P* < 0.001. ***** P* < 0.0001.


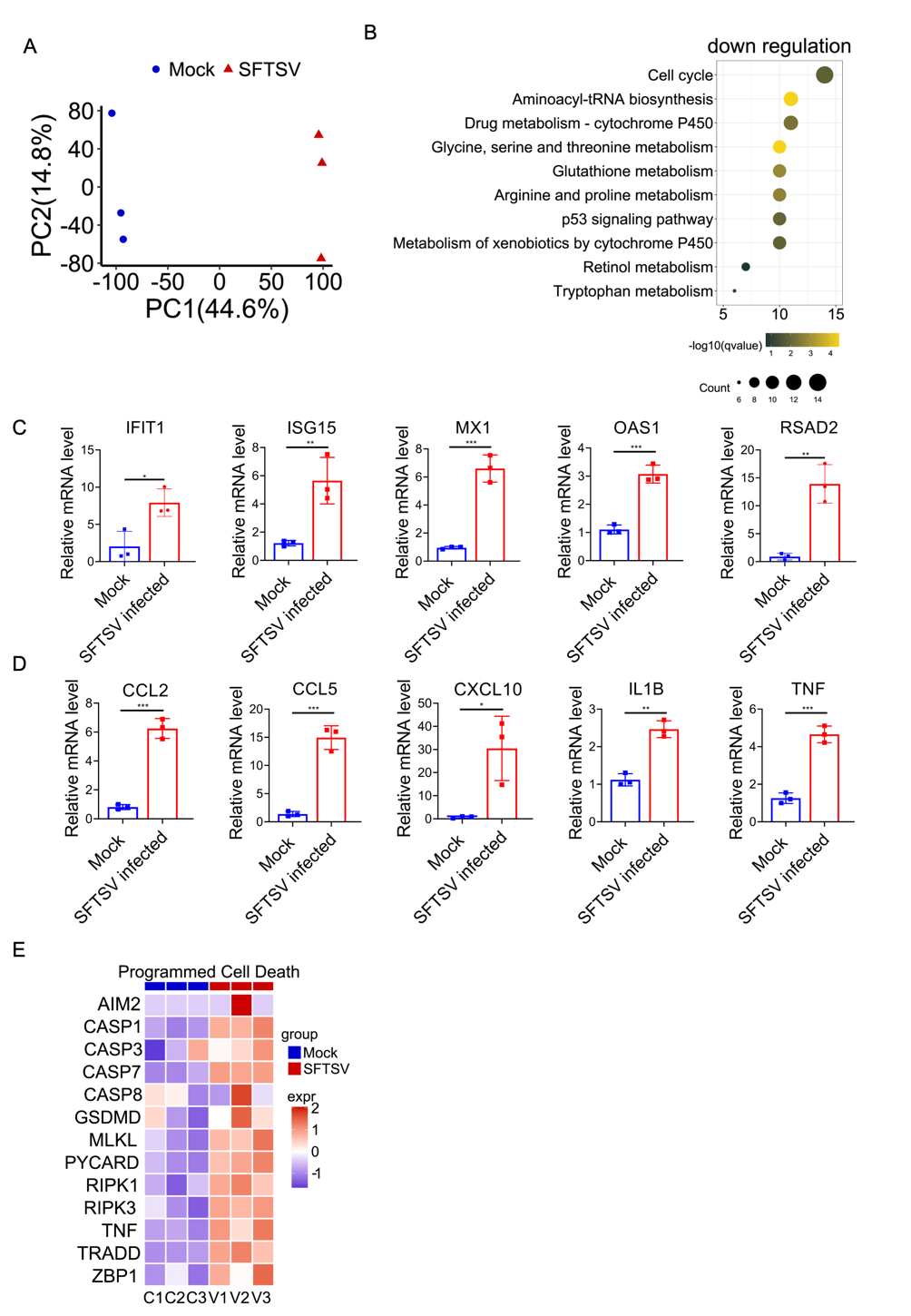


Figure S3 Transcriptomic Characteristics of SFTSV Infection in Pancreatic Organoids

(A) PCA reveals transcriptomic differences between mock group (n=3) and SFTSV-infected group (n=3). (B) The bubble plot shows the top 10 terms in the KEGG analysis of downregulated genes in pancreatic organoids of SFTSV infection. ISGs induction (C) and proinflammatory cytokines production (D) were confirmed by qRT-PCR in human pancreatic organoids. (E) The heatmap shows the expression of representative programmed cell death-related genes in pancreatic organoids of SFTSV infection. Data shown are means ± SEM. Statistical significance was analyzed by Student’s t-test. **P* < 0.05; ***P* < 0.01; ****P* < 0.001. ***** P* < 0.0001.


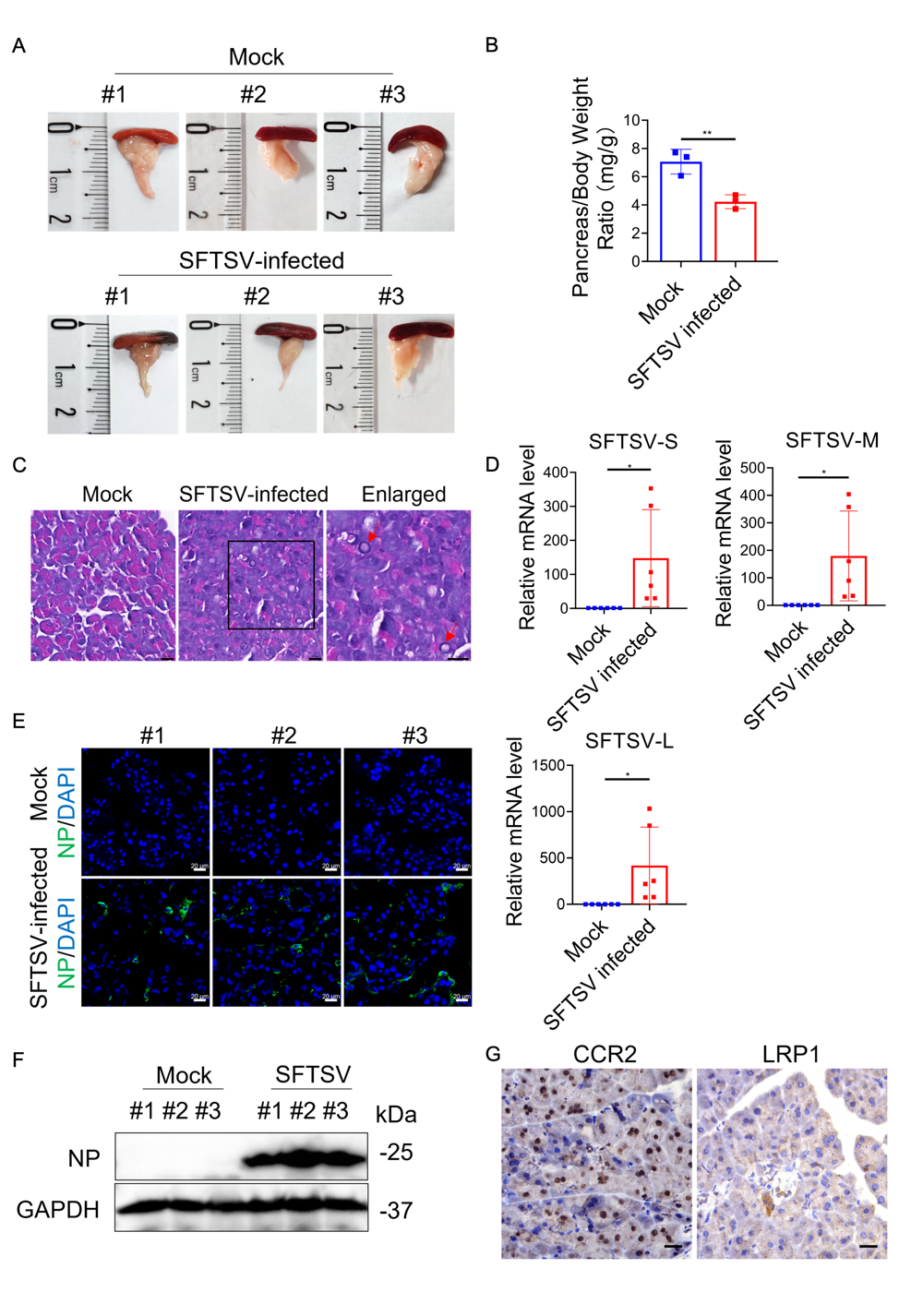


Figure S4 Direct infection of murine pancreatic tissue by SFTSV

(A) Gross morphology showing pancreatic atrophy in mock group (n=3) and SFTSV-infected group (n=3). (B) Quantification of pancreas to body weight ratio. (C) H&E staining of pancreatic sections showing viral inclusion body-like structures (red arrows). Scale bar, 20 μm. (D) Relative mRNA expression levels of SFTSV genomic segments (S, M, L) by qRT-PCR. (E) Immunofluorescence detection of viral NP expression (green) in murine pancreatic tissue. Scale bars, 20 μm. (F) Western blot detection of SFTSV NP expression (25 kDa) in murine pancreatic tissues. (G) Immunohistochemical detection of SFTSV receptors CCR2 and LRP1 expressions in murine pancreatic tissues. Scale bar, 20 μm. Experiment was performed in triplicates. Data shown are means ± SEM. Statistical significance was analyzed by Student’s t-test. **P* < 0.05; ***P* < 0.01; ****P* < 0.001; ***** P* < 0.0001.


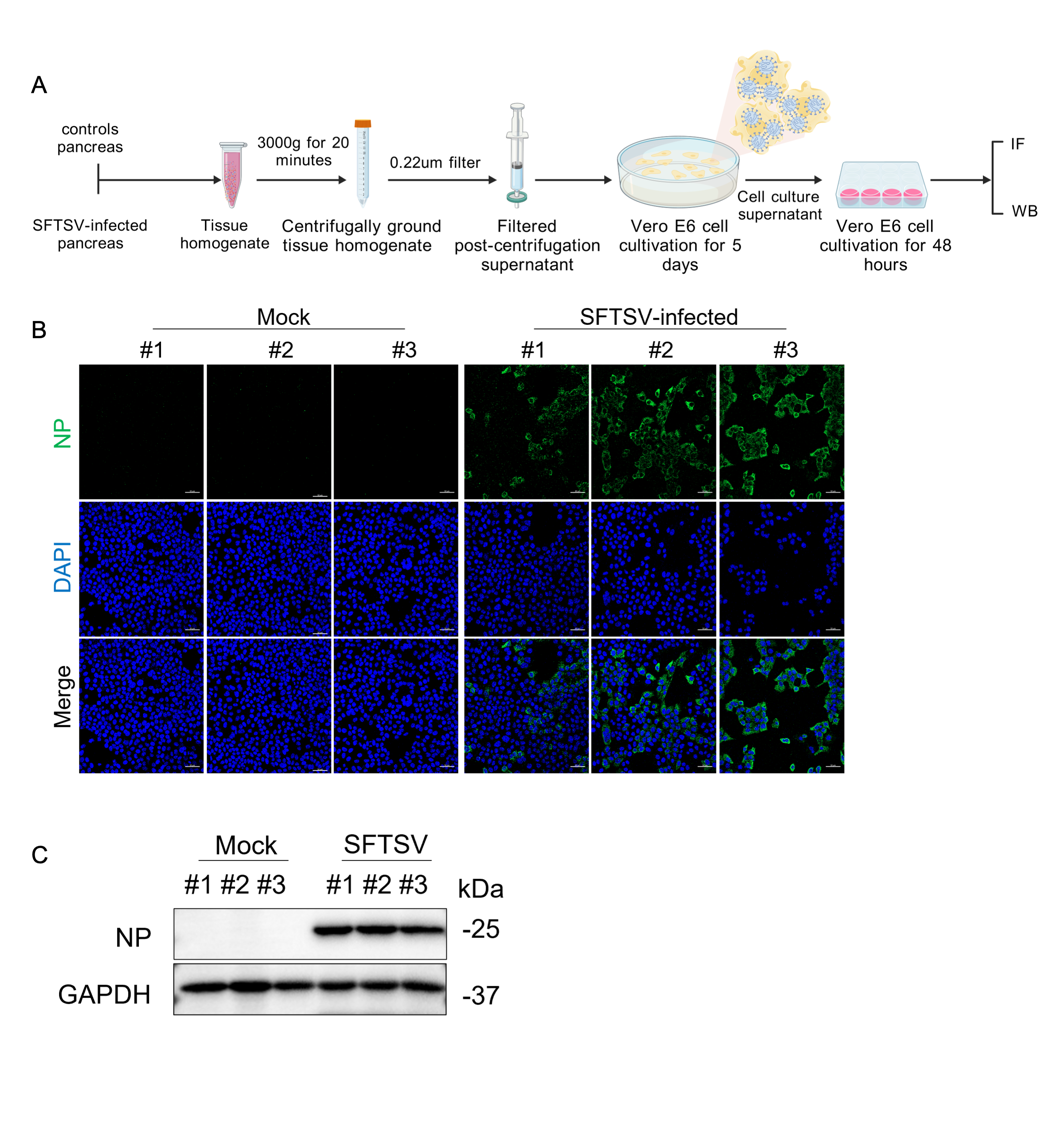


Figure S5 Virus was isolated from SFTSV-infected mouse pancreas

(A) Schematic of viral isolation from pancreatic homogenates. Pancreatic homogenates (10% penicillin and streptomycin in PBS(v/v)) were centrifuged, filtered, and used to inoculate Vero E6 monolayers, with three biological replicates per group. After 5 d, supernatants were passaged onto new Vero E6 cells for 48 h (created with BioGDP.com). (B) Immunofluorescence detection of viral NP expression (green) in Vero E6 cells with supernatants culture. Scale bars, 50 μm. (C) Western blot detection of SFTSV NP expression in Vero E6 cells with supernatants culture.


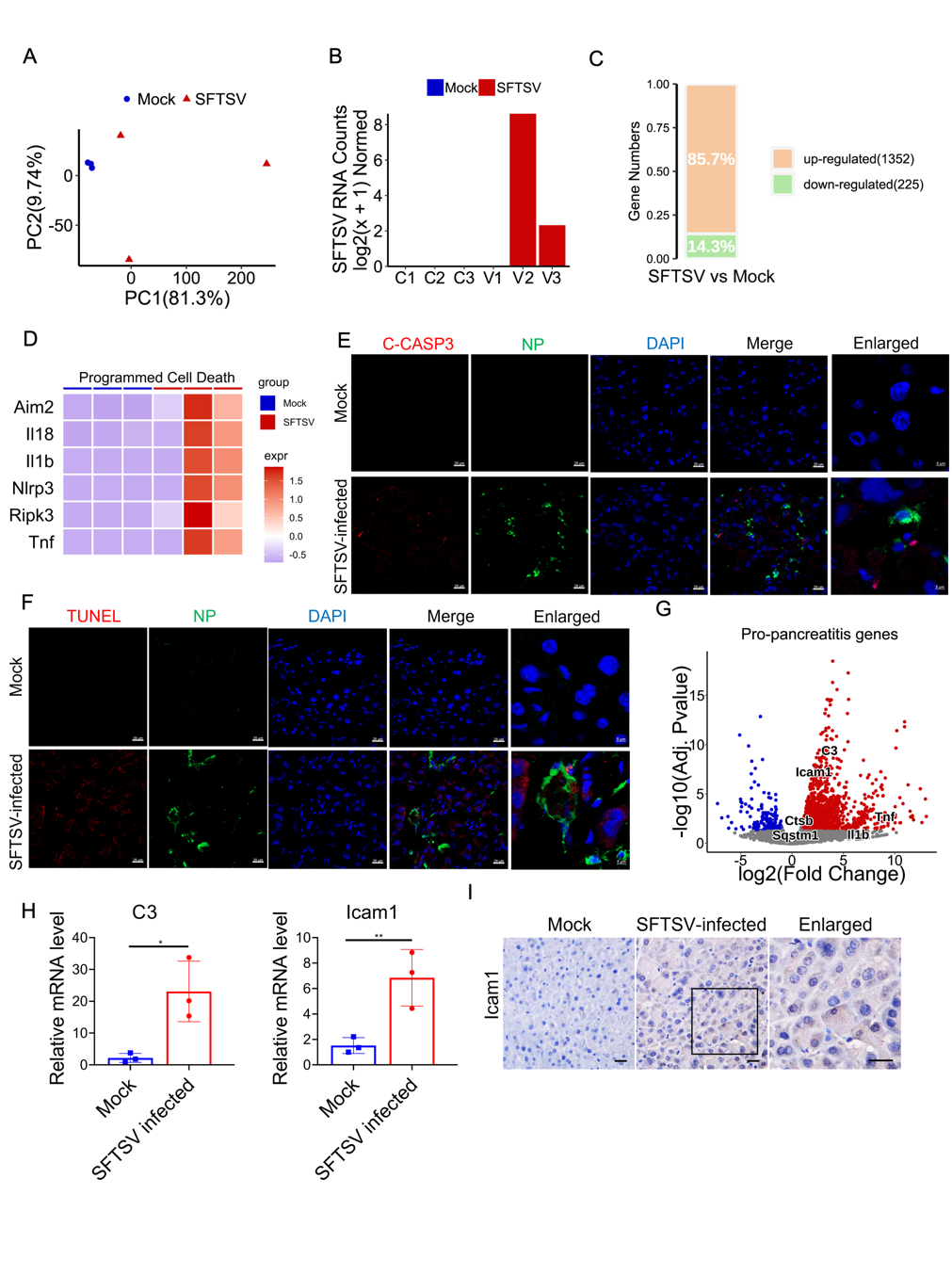


Figure S6 Transcriptome characteristics of the pancreas of SFTSV-infected mice

(A) PCA reveals transcriptomic differences between mock group (n=3) and SFTSV-infected group (n=3). (B) Bar plot shows the counts of SFTSV genomic fragments detected in RNA-seq of murine pancreatic tissues. (C) The stacked plot shows the direction and proportion of DEGs of pancreatic tissues RNA-seq from SFTSV-infected versus mock groups. (D) The heatmap shows the expression of representative programmed cell death-related gene in murine pancreatic tissue of SFTSV infection. (E) Immunofluorescence detection of viral NP expression (green), C-CASP3 expression (red) in murine pancreatic tissue. Scale bars, 20 μm and 5 μm in the enlarged image. (F) Immunofluorescence detection of TUNEL^+^ apoptotic cells (red) and viral NP expression (green) in murine pancreatic tissues. Scale bars, 20 μm and 5 μm in the enlarged image. (G) The volcano plot shows the expression of pro-pancreatitis genes in RNA-seq of pancreatic tissues. (H) qRT-PCR validation of *C3* and *Icam1* expression levels from the pro-pancreatitis genes volcano plot in murine pancreatic tissues. (I) Immunohistochemical detection of Icam1 in murine pancreatic tissues. Scale bar, 20 μm. Data shown are means ± SEM. Statistical significance was analyzed by Student’s t-test. **P* < 0.05; ***P* < 0.01; ****P* < 0.001; ***** P* < 0.0001. Experiment was performed in triplicates


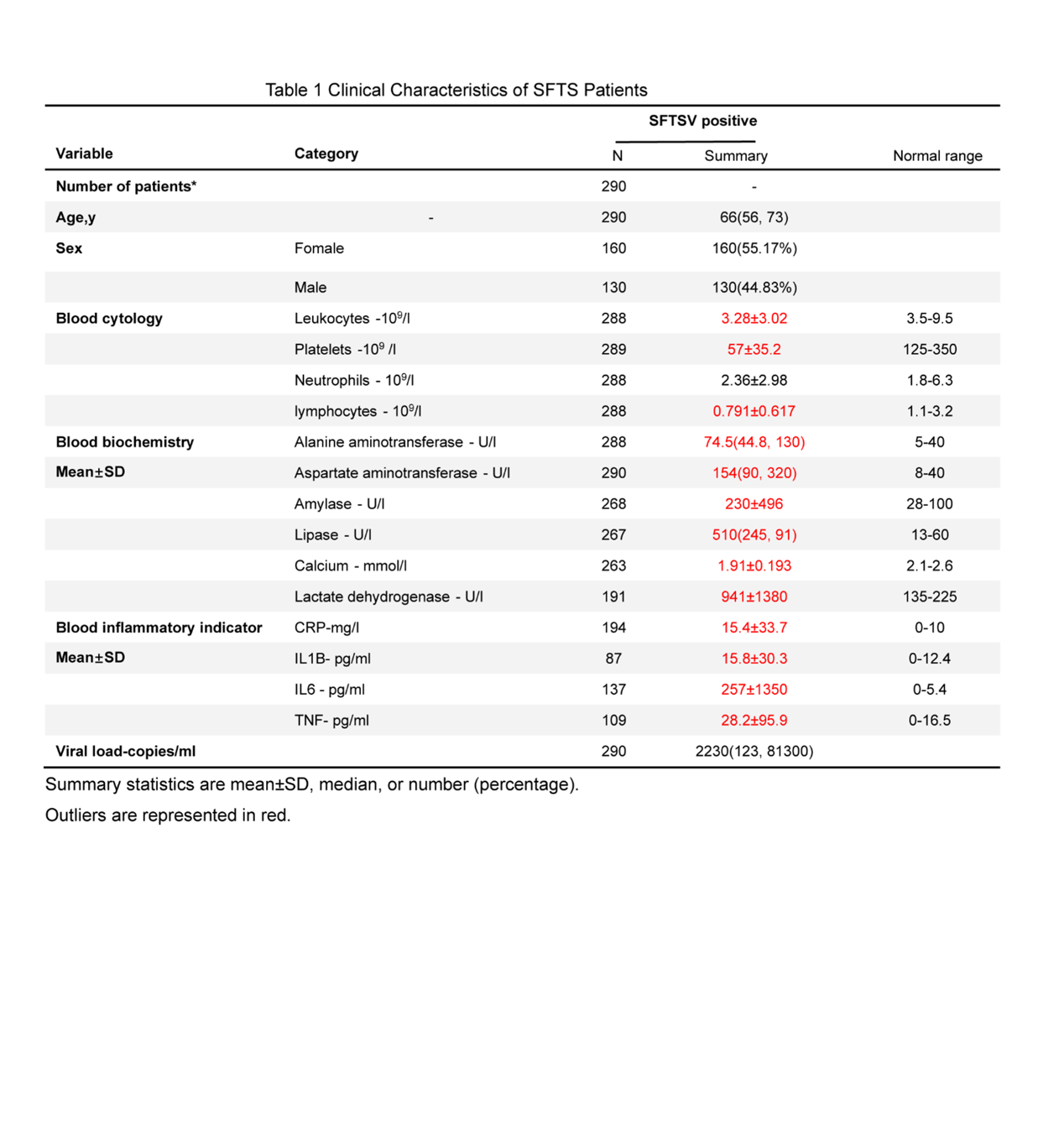


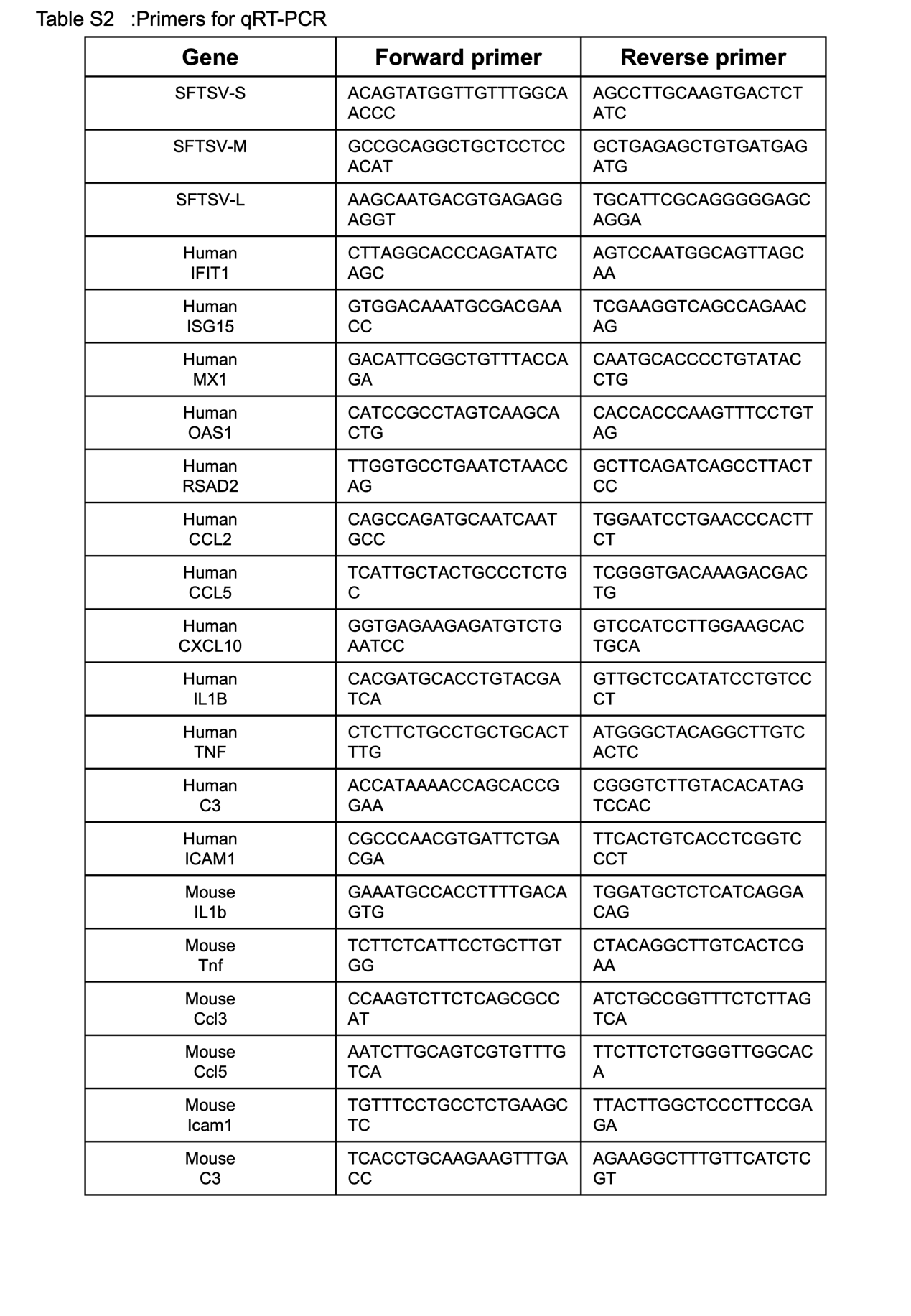

Supplement: Supplementary file 1 — Supporting Information [file ADVS-13-e15862-s001.docx]
